# Host regulation of liver fibroproliferative pathology during experimental schistosomiasis via interleukin-4 receptor alpha

**DOI:** 10.1371/journal.pntd.0005861

**Published:** 2017-08-21

**Authors:** Justin Komguep Nono, Hlumani Ndlovu, Nada Abdel Aziz, Thabo Mpotje, Lerato Hlaka, Frank Brombacher

**Affiliations:** 1 Cytokines and Diseases Group, International Centre for Genetic Engineering and Biotechnology, Cape Town Component, Cape Town, South Africa; 2 Division of Immunology, Health Science Faculty, University of Cape Town & Immunology of Infectious Disease Research Unit, South African Medical Research Council (SAMRC), Cape Town, South Africa; 3 The Medical Research Centre, Institute of Medical Research and Medicinal Plant Studies (IMPM), Ministry of Scientific Research and Innovation, Yaoundé, Cameroon; 4 Department of Integrative Biomedical Sciences, Health Sciences Faculty, University of Cape Town, Cape Town, South Africa; 5 Department of Chemistry, Faculty of Science, Cairo University, Giza, Egypt; Uniformed Services University, UNITED STATES

## Abstract

Interleukin-4 receptor (IL-4Rα) is critical for the initiation of type-2 immune responses and implicated in the pathogenesis of experimental schistosomiasis. IL-4Rα mediated type-2 responses are critical for the control of pathology during acute schistosomiasis. However, type-2 responses tightly associate with fibrogranulomatous inflammation that drives host pathology during chronic schistosomiasis. To address such controversy on the role of IL-4Rα, we generated a novel inducible IL-4Rα-deficient mouse model that allows for temporal knockdown of *il-4rα* gene after oral administration of Tamoxifen. Interrupting IL-4Rα mediated signaling during the acute phase impaired the development of protective type-2 immune responses, leading to rapid weight loss and premature death, confirming a protective role of IL-4Rα during acute schistosomiasis. Conversely, IL-4Rα removal at the chronic phase of schistosomiasis ameliorated the pathological fibro-granulomatous pathology and reversed liver scarification without affecting the host fitness. This amelioration of the morbidity was accompanied by a reduced Th2 response and increased frequencies of FoxP3^+^ Tregs and CD1d^hi^CD5^+^ Bregs. Collectively, these data demonstrate that IL-4Rα mediated signaling has two opposing functions during experimental schistosomiasis depending on the stage of advancement of the disease and indicate that interrupting IL-4Rα mediated signaling is a viable therapeutic strategy to ameliorate liver fibroproliferative pathology in diseases like chronic schistosomiasis.

## Author summary

Liver fibroproliferative diseases drive a considerable fraction of the overall human mortality. This is closely linked to the absence of efficient control measures against such diseases. Schistosomiasis, a chronic disease that affects humans, preferentially causes liver fibrosis and is responsible for devastating economic losses in developing nations where the disease is still endemic. Using reverse genetics, loss-of-function mouse models have helped uncover a protective role for Interleukin-4 receptor (IL-4Rα) in the host survival to experimental schistosomiasis. However, given the contributing role for this receptor in the etiology of some models of tissue fibrosis, its role during chronic schistosomiasis where the highly fibrotic liver of the infected individuals mediate the morbidity had not been properly addressed hitherto. Taking advantage of a third generation mouse model of inducible loss of a gene, we found a debilitating role for IL-4 receptor during chronic schistosomiasis as signaling via this receptor supported both liver inflammation and fibrosis. These findings demonstrate that although the host requires IL-4Rα to survive the acute phase of schistosomiasis, the more clinically relevant morbid phase of the disease is driven by the excessive utilization of this receptor. A therapeutic potential of blocking IL-4Rα to ameliorate liver fibroproliferative disease is therefore suggested.

## Introduction

Schistosomiasis is a parasitic disease caused by blood-dwelling parasitic flatworms of the genus *Schistosoma*, mainly, *Schistosoma mansoni* (*S*. *mansoni)*, *S*. *japonicum* and *S*. *haematobium* that are infective to humans and the most clinically relevant [1]. Schistosomiasis is estimated to affect more than 200 million people worldwide and causes up to 200,000 deaths per annum in developing countries [1]. The disease is caused by parasite eggs trapped in the microvasculature of the host organs (liver, intestine and bladder) that induce a vigorous inflammatory response [1]. The kinetics of the ensuing immune responses induced by *S*. *mansoni* infection are well defined and characterized [2,3]. Briefly, the outcomes of disease persistence and progression are organ enlargement, fibrosis, scarring, portal hypertension or hematuria (*S*. *haematobium* specifically) that drive host morbidity and eventually death in severe cases [1].

The immune response to schistosomiasis, similarly to that against other tissue-dwelling helminth infections [4–6], is highly polarized as it progresses, going from i) an early Th1 response to ii) a powerful Th2 response that culminates as the adult parasite-released eggs are trapped in the host tissues [2,3] and finally iii) a chronic regulatory phase with a minimized but still dominant Th2 response [3,7,8] with a more clinically relevant tissue fibroproliferative pathology. Our current understanding of schistosomiasis pathology heavily relies on the use of experimental murine models [2]. Studies aimed at uncovering factors that drive host protection or susceptibility to schistosomiasis have been conducted using gene-deficient mice. The disease associates with the formation of granulomas and excessive collagen deposition (fibrosis) around tissue-trapped eggs [3,7,8]. An important role was defined for the host immune effector responses in these pathognomonic processes as nude mice [9], T cell-depleted [10–13] or mice with severe combined immunodeficiency [14] failed to form proper fibrogranulomatous responses. Even though Th1, Th17 and Treg responses have been shown to play major roles in regulating schistosomiasis pathogenesis, type 2 immune responses, which are typically induced by the disease-mediating eggs of the parasite [15–17], have been ascribed a more dominant role [3,7,8,18].

Initiation and polarization of type 2 immune responses is orchestrated by interleukin-4 (IL-4) and IL-13 signaling via a common IL-4Rα chain [2,19]. Signaling via this receptor drives the activation of the transcription factor STAT6 in hematopoietic cells, the proliferation of T and B cells, the production of immunoglobulins by B cells, the priming and chemotaxis of mast cells and basophils [2,19]. In non-hematopoietic cells, this receptor plays a central role in inducing airway hyper-responsiveness by enhancing contractions and mucus secretion by gut epithelial cells [20] and has been shown to play a role in STAT6-dependent fibroblast activation leading to collagen deposition that define fibro-proliferative diseases [21,22]. Understandably, mice deficient in this receptor show impaired granuloma formation, enhanced liver damage and augmented gut inflammation that leads to endotoxemia and septic shock during acute schistosomiasis [23–26]. Moreover, studies conducted in our laboratory have refined the requirement of IL-4Rα to a cell-specific level showing that IL-4Rα-responsive macrophages [25], pan-T cells [27] and smooth muscles cells [28] are individually essential for driving host survival and limiting tissue pathology during acute schistosomiasis.

In all these studies employing mice constitutively deficient in IL-4Rα, a critical role for IL-4Rα mediated signaling during acute [25,26] and chronic schistosomiasis [25,29,30] is suggested. However, the constitutive lack of IL-4Rα led such transgenic mice to succumb prematurely to experimental schistosomiasis with high (acute model) as well as low (chronic model) infection doses i.e. chronic model of infections succumb during the acute phase in the absence of IL-4Rα [30] casting an equivoque on the reliability of using models of constitutive deletion of IL-4Rα to assess the role of this receptor during chronic schistosomiasis. Moreover, congenital IL-4Rα deletion has now been shown to affect the development of animals [31], challenging our current knowledge on the role of IL-4Rα throughout experimental schistosomiasis (acute and chronic) using mouse models of constitutive IL-4Rα deficiency.

In this study, the role of IL-4Rα during acute and chronic schistosomiasis was investigated using a novel murine model that allows for inducible deletion of *il-4rα* gene at any time point during *S*. *mansoni* infection. Our findings further confirmed a protective role played by IL-4Rα mediated signaling during acute schistosomiasis. Contrastingly, we showed for the first time that partial deletion of the *il-4rα* gene, specifically, at the chronic stage of schistosomiasis ameliorates the tissue pathology by reducing type-2 immune responses, improving immune balance between T helper cytokines and skewing the diminished immune response towards a more regulatory profile without affecting animal viability.

## Results

### Generation and characterization of RosaCreER^T2-/+^IL-4Rα^-/lox^ mice

**Fig 1:**
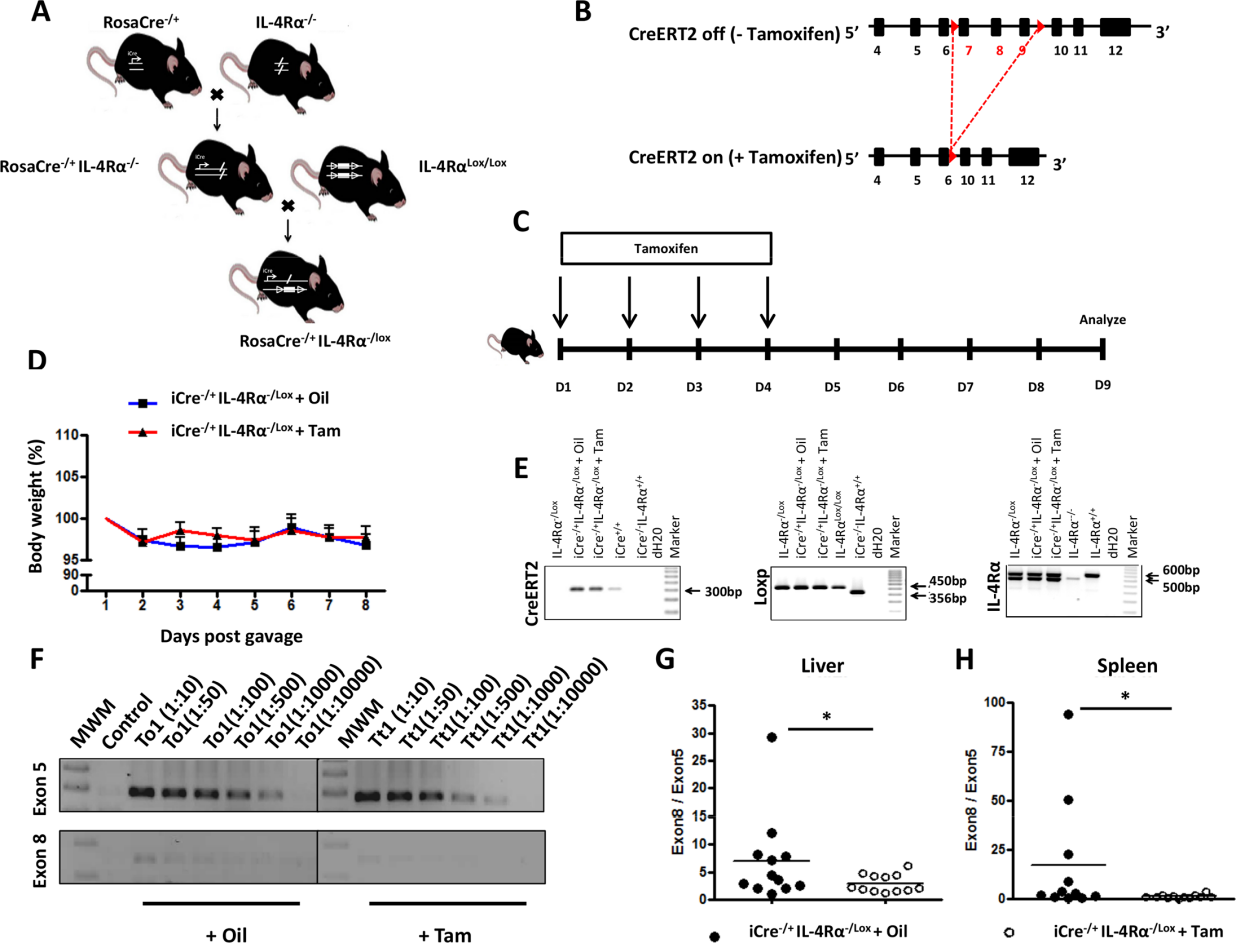
Generation and genotypic characterization of the RosaCre^-/+^ IL-4Rα^-/lox^ deletable mouse model. **A.** IL-4Rα^-/-^ C57BL/6 mice were intercrossed with RosaCre expressing and IL-4Rα^Lox/Lox^ mice to generate RosaCre^-/+^ IL-4Rα^-/lox^ (i.e. iCre^-/+^ IL-4Rα^-/lox^ mice). **B.** Schematic of the expected *il-4r* gene loci in iCre^-/+^ IL-4Rα^-/lox^ mice fed or not with Tamoxifen. **C.** Experimental design for assessing the inducible deletion of *il-4rα* in Tamoxifen-fed iCre^-/+^ IL-4Rα^-/lox^ mice. **D.** Short-term effect of Tamoxifen gavage on body weight. **E.** Genotyping of iCre^-/+^ IL-4Rα^-/lox^ mice. The *creer*^*T2*^ specific amplicon is 300bp, *loxp* is 450bp. In wild type mice, the *il-4rα* amplicon is 600bp and 471bp in mice with a deleted *il-4rα* gene. **F.** Semi-quantitative PCR analyses of Exon 8 (only deleted in mice with an activated CreER^T2^) vs. Exon 5 (present in all mice) between iCre^-/+^ IL-4Rα^-/lox mice^ fed either with oil or Tamoxifen. (**G, H).** Quantitative real-time PCR of exon 8 over exon 5 from the *il-4rα* gene. Genomic DNA was extracted from liver tissue (**G**) or splenocytes (**H**) and exon 8 from *il-4rα* was quantified by qPCR and normalized to exon 5. Each experiment was conducted at least twice with 3–4 mice per group. Data are expressed as mean ± SD; NS = p > 0.05; * = p < 0.05; ** = p < 0.01; *** =, p < 0.001; **** = p < 0.0001.

Inducible IL-4Rα deficient C57BL/6 mice (RosaCreER^T2^IL-4Rα^-/lox^ mice, termed i^Cre-/+^IL-4Rα^-/lox^ mice) were established using a modified cyclization recombinase (Cre) under the control of the ubiquitously expressed *Rosa* promoter. This modified Cre incorporated a mutated fragment of the ligand-binding domain of the estrogen receptor (ER^T2^), that makes the activity of Cre conditional to the specific presence of Tamoxifen, an estrogen ligand homologue [32]. RosaCreER^T2^ C57BL/6 mice were intercrossed with IL-4Rα^-/-^ C57BL/6 mice [33] to generate RosaCreER^T2^IL-4Rα^-/-^ mice (Fig 1A) and subsequently intercrossed with floxed IL-4Rα (IL-4Rα^lox/lox^) C57BL/6 mice (exon 6 to 8 flanked by loxP) (Fig 1B, [25]) to generate RosaCreER^T2-/+^IL-4Rα^-/lox^ C57BL/6 mice (Fig 1A). Tamoxifen feeding (Fig 1C) did not impair the fitness of naïve RosaCreER^T2-/+^IL-4Rα^-/lox^ C57BL/6 mice, as judged by body weight change (Fig 1D). In Tamoxifen-fed RosaCreER^T2-/+^IL-4Rα^-/lox^ C57BL/6 mice, CreER^T2^-mediated deletion of the exon 6 to 8 of the *il-4rα* gene (Fig 1B) was identified by specific *Cre*-, *loxp*- and *il-4rα*- PCR genotyping from tail DNA (Fig 1E), and real-time qPCR from liver (Fig 1G) and spleen DNA (Fig 1H).

**Fig 2:**
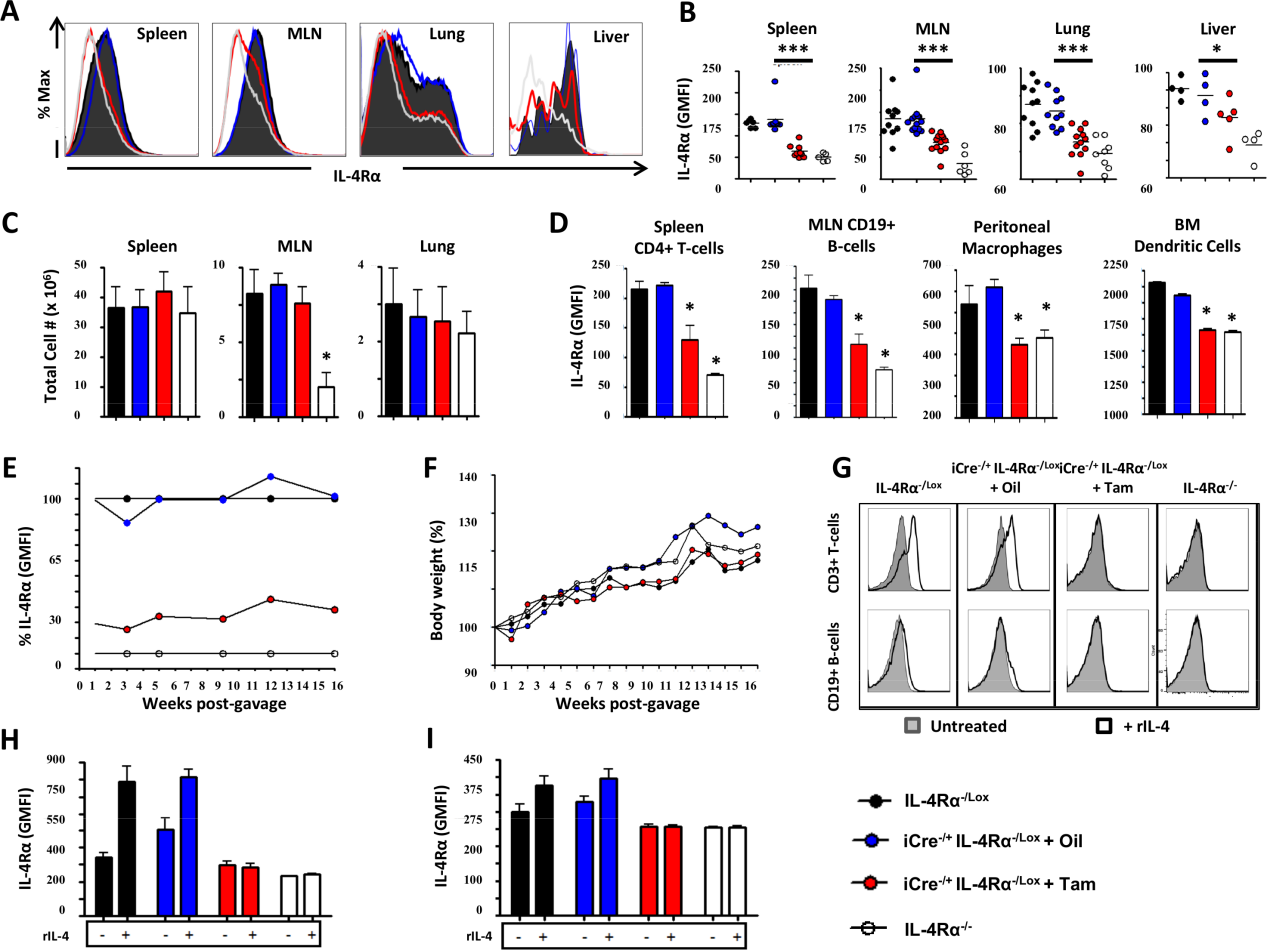
Phenotypic and functional characterization of the RosaCre^-/+^ IL-4Rα^-/lox^ deletable mouse model. **A.** IL-4Rα GMFI of total cells from several organs 5 days following Tamoxifen administration summarized in **B. C.** Total cell numbers in several organs. **D.** IL-4Rα GMFI of major immune cells taken from several organs. **E.** Kinetics of IL-4Rα GMFI change over time following Tamoxifen treatment of RosaCre^-/+^ IL-4Rα^-/lox^ mice. A relative IL-4Rα expression of 100% was assigned to IL-4Rα^-/lox^ mice and a relative expression of 0% was assigned to IL-4Rα^-/-^ mice. Do note the similarity in IL-4Rα expression levels between IL-4Rα^-/lox^ and oil-treated RosaCre^-/+^ IL-4Rα^-/lox^ control mice. **F.** Long-term effect of Tamoxifen gavage on the body weight of RosaCre^-/+^ IL-4Rα^-/lox^ mice. **G.** Upregulation of surface expression of IL-4Rα by spleen lymphoid cells stimulated with rIL-4. Cells were collected 48 hours following incubation with rIL-4 and IL-4Rα mean surface expression on CD3^+^ T (**H**) and CD19^+^ B cells (**I**) was determined and plotted. Each experiment was conducted at least twice with 3–6 mice per group. Data are expressed as mean ± SD; NS = p > 0.05; * = p < 0.05; ** = p < 0.01; *** =, p < 0.001; **** = p < 0.0001.

Analysis of IL-4Rα surface expression on total cells from different organs by flow cytometry (Fig 2A) demonstrated that IL-4Rα was considerably depleted following administration of Tamoxifen to RosaCreER^T2-/+^IL-4Rα^-/lox^ mice (Fig 2A and 2B and S1A Fig). To rule out a non-specific toxic effect or bystander immune alteration in Tamoxifen-fed RosaCreER^T2-/+^IL-4Rα^-/lox^ mice, spleen weights (S1B Fig), organ cellularity (Fig 2C and S1C Fig), seric liver enzymes (S1D Fig), baseline IgE levels (S1E Fig), IL-2-driven proliferative responses of total splenocytes (S1F Fig), frequencies of major myeloid and lymphoid cells (S2A Fig and S2B Fig) and total CD4^+^ (S2C Fig) and CD8^+^ (S2D Fig) T cell numbers in spleens and mesenteric lymph nodes (MLN) were determined. This revealed that, amid a minimal cellular deficiency in Spleen CD4^+^ and CD8^+^ T cells at baseline in our murine model, organ cellularity, weight and baseline cellular and humoral immune responses were not generally affected in Tamoxifen-fed RosaCreER^T2-/+^IL-4Rα^-/lox^ mice (Fig 2C, S1 Fig and S2 Fig). Tamoxifen treatment of RosaCreER^T2-/+^IL-4Rα^-/lox^ mice significantly reduced or even abrogated surface IL-4Rα expression on spleen CD4^+^ T cells, MLN CD19^+^ B cells, peritoneal macrophages as well as bone marrow-derived dendritic cells (Fig 2D). Robustness of IL-4Rα knockdown in Tamoxifen-fed RosaCreER^T2-/+^IL-4Rα^-/lox^ mice was monitored in white blood cells over a period of 16 weeks following Tamoxifen administration (Fig 2E). A relative expression level of 0% was attributed at all times to blood B cells from global IL-4Rα^-/-^ mice, whereas a relative expression level of 100% was attributed to IL-4Rα^-/lox^ mice. Blood B cells from oil-fed RosaCreER^T2-/+^IL-4Rα^-/lox^ mice oscillated around a level of IL-4Rα expression of 100%, Tamoxifen-fed RosaCreER^T2-/+^IL-4Rα^-/lox^ mice expressed only 20% of IL-4Rα (Fig 2E), which increased to a maximum of 30% in 16 weeks with no significant body weight changes throughout the monitoring period (Fig 2F).

To assess the cellular knockdown of IL-4Rα functionally, splenocytes from IL-4Rα^-/lox^ littermate controls, oil-fed RosaCreER^T2-/+^IL-4Rα^-/lox^, Tamoxifen-fed RosaCreER^T2-/+^IL-4Rα^-/lox^ and IL-4Rα^-/-^ mice were cultured with or without recombinant IL-4 for 48h then harvested, stained for IL-4Rα expression and analyzed by flow cytometry (Fig 2G). As expected, spleen T (Fig 2H) and B cells (Fig 2I) derived from IL-4Rα^-/lox^ mice and oil-fed RosaCreER^T2-/+^IL-4Rα^-/lox^ controls up-regulated IL-4Rα expression after the addition of IL-4 (Fig 2G). In contrast, rIL-4 stimulated spleen T and B cells derived from Tamoxifen-fed RosaCreER^T2-/+^IL-4Rα^-/lox^ or from IL-4Rα^-/-^ mice showed no upregulation of IL-4Rα expression (Fig 2H and 2I). This showed functional impairment of IL-4Rα mediated signaling on cells from Tamoxifen-fed RosaCreER^T2-/+^IL-4Rα^-/lox^ mice, complementing the above-demonstrated genotypic and phenotypic impairments. Taken together, these results indicated that Tamoxifen administration to the RosaCreERT2^-/+^IL-4Rα^-/lox^ mouse model leads to a timely, efficient, safe and stably induced IL-4Rα knockdown mouse model.

### Host survival requires IL-4Rα mediated signaling during acute but not chronic schistosomiasis

**Fig 3:**
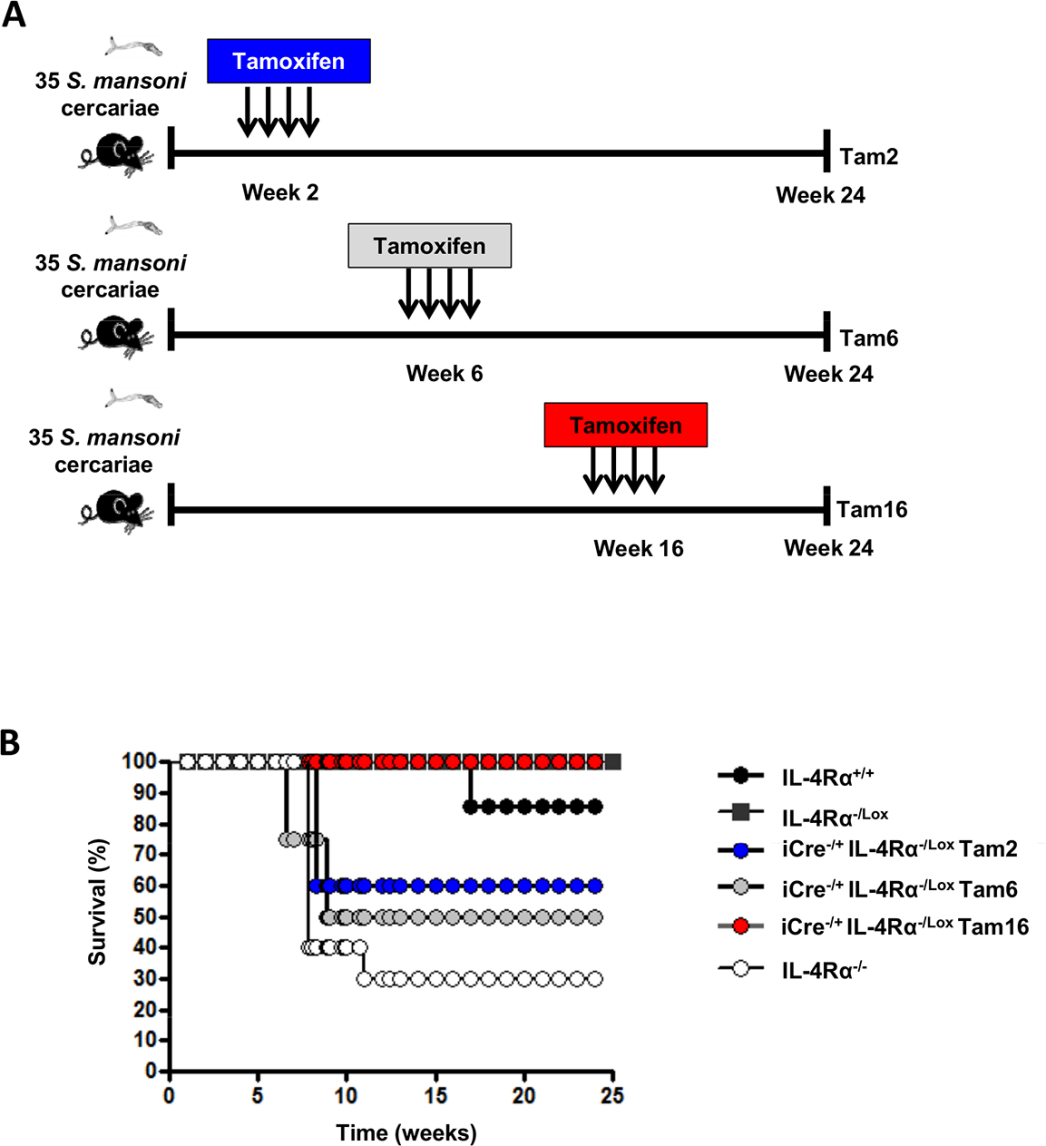
Different phenotypes after knocking down IL-4Rα at different phases of experimental schistosomiasis. **A.** Experimental design. **B.** Survival curve representing the cumulative profile from 2 different infections (n = 5–10 mice).

A protective role for IL-4Rα mediated signaling has been established during acute schistosomiasis, where IL-4Rα deficient mice but not wild-type (wt) mice died around 6 to 8 weeks after natural infection with *S*. *mansoni* [25]. To determine whether IL-4Rα mediated signaling is required throughout the course of experimental schistosomiasis, IL-4Rα was knocked down in *S*. *mansoni*-infected RosaCreER^T2-/+^IL-4Rα^-/lox^ mice at the early acute (Tamoxifen administration at 2 weeks post-infection termed Tam2), late acute (Tamoxifen administration at 6 weeks post-infection termed Tam6) and chronic phase (Tamoxifen administration at 16 weeks post-infection termed Tam16) (Fig 3A), as previously defined [3]. As expected, most of IL-4Rα deficient mice (70%) succumbed prematurely to infection with 35 *S*. *mansoni* cercariae as early as from 7 weeks post-infection (Fig 3B). Similarly, the viability of Tam2- and Tam6-fed RosaCreER^T2-/+^IL-4Rα^-/lox^ mice declined rapidly (60 and 50% respectively at week 8 post-infection). From Tam-2-fed, Tam-6 fed or IL-4Rα deficient mice, no death was further reported as from 12 weeks post-infection, at the chronic phase of the disease. This indicated that IL-4Rα is necessary for host survival during acute schistosomiasis, but not required for host survival at the chronic phase of the disease. Indeed, removal of IL-4Rα in Tam16-fed *S*. *mansoni*-infected RosaCreER^T2-/+^IL-4Rα^-/lox^ mice failed to affect the morbidity (as indicated by serum levels of alanine transaminase as a marker of liver disease (S3 Fig) and the mortality (Fig 3B) up to 24 weeks post-infection, further supporting a dispensable role of IL-4Rα mediated signaling during chronic schistosomiasis. Taken together our results suggest that IL-4Rα mediated signaling differentially regulates schistosomiasis disease depending on the stage of the infection.

### Early IL-4Rα knockdown before egg production (week 2) exacerbates acute schistosomiasis

**Fig 4:**
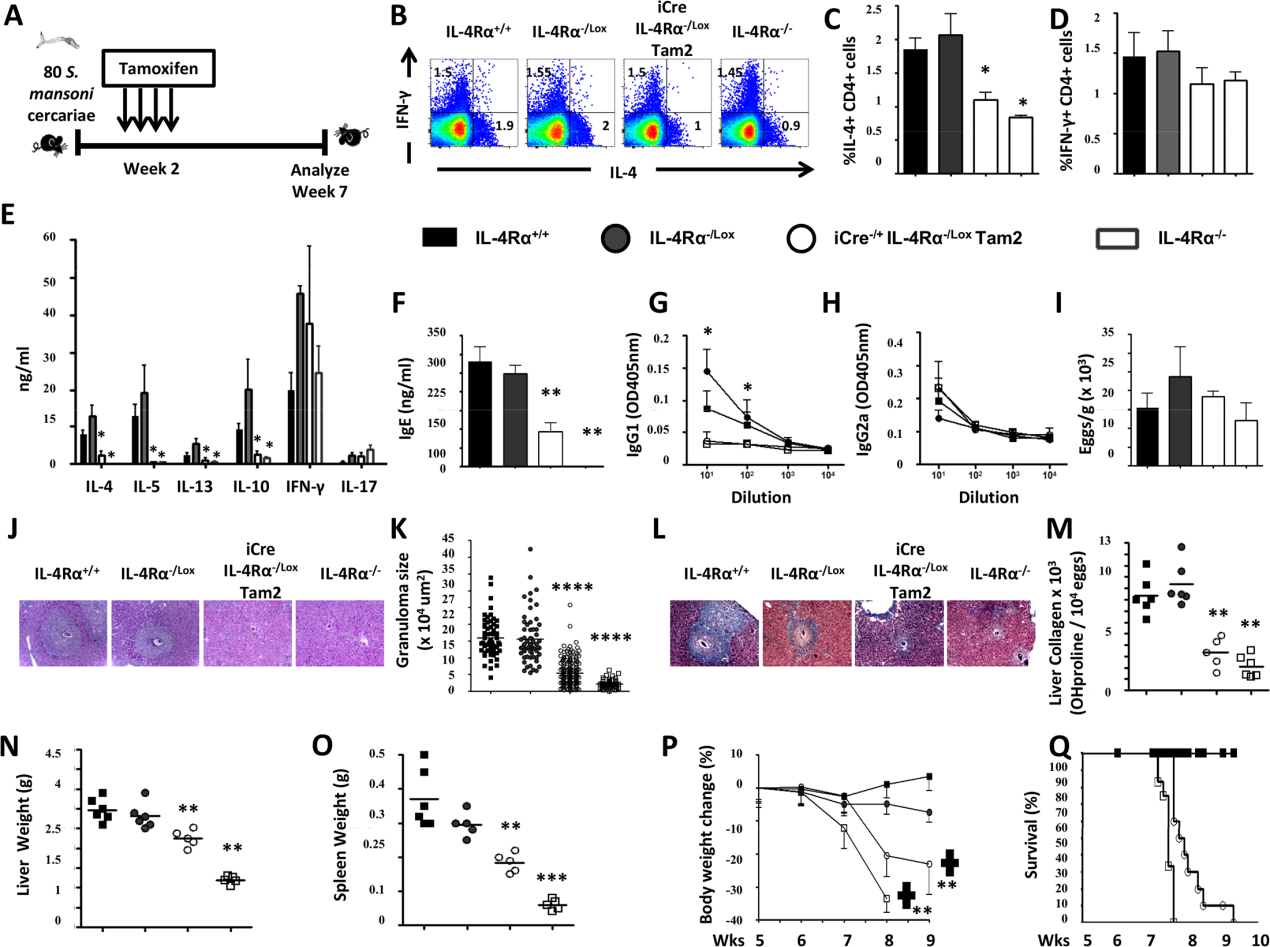
Immunological and histopathological profile of *S*. *mansoni-*infected mice after knocking down IL-4Rα 2 weeks post-infection. **A.** Experimental design. **B.** Representative plot of MLN cytokine-producing CD3^+^CD4^+^ T cell frequencies after stimulation with PMA/Ionomycin/Monensin cocktail. Summaries of IL-4-producing (**C**) and IFNγ-producing (**D**) CD3^+^CD4^+^ T cell frequencies from 2 independent experiments conducted with 3–8 mice are shown. **E.** Cytokine release detected by ELISA in the supernatant of anti-CD3 stimulated MLN cells. **F.** Total seric IgE in *S*. *mansoni*-infected mice. SEA-specific seric IgG1 (**G**) and IgG2a (**H**) isotype antibodies. **I.** Liver Egg burden. **J.** Formalin-fixed Hematoxylin/Eosin-stained sections of liver tissue from infected animals for morphological analyses (displayed here at 100X). **K.** Area sizes of egg-surrounding granuloma are computed. **L.** Formalin-fixed CAB-stained sections of liver tissue from infected animals for collagen detection (displayed here at 100X). **M.** Hydroxyproline content measured by colorimetry is displayed as a measure of tissue collagen content. Liver (**N**) and Spleen (**O**) weights. **P.** Body weight change over time following *S*. *mansoni* infection. **Q.** Survival curve following *S*. *mansoni* infection. Each experiment was conducted at least twice with 5–10 mice per group. Data are expressed as mean ± SD; NS = p > 0.05; * = p < 0.05; ** = p < 0.01; *** =, p < 0.001; **** = p < 0.0001.

The findings above demonstrated an impaired viability of *S*. *mansoni*-infected mice following IL-4Rα knockdown at 2 weeks post-infection (Tam2). Hence, the immune and histopathological response of Tam2-fed RosaCreER^T2-/+^IL-4Rα^-/lox^ mice (termed iCre^-/+^IL-4Rα^-/lox^ Tam2, Fig 4A), which might associate with the host premature death during experimental schistosomiasis was dissected. A consistent reduction of MLN CD4^+^ (S4A Fig) and CD8^+^ (S4B Fig) T cell counts was observed in *S*. *mansoni*-infected i^Cre-/+^IL-4Rα^-/lox^ Tam2 animals at week 7 when compared to littermate controls, consistent with the *S*. *mansoni*-infected global IL-4Rα^-/-^ animals (S4A Fig and S4B Fig). *Ex vivo* stimulation with a cocktail of PMA/Ionomycin/Monensin for 4h at 37°C and subsequent intracellular FACS analysis of MLN cells from *S*. *mansoni*-infected i^Cre-/+^IL-4Rα^-/lox^ Tam2 animals at week 7 resulted in impaired IL-4 production, but similar IFNγ production when compared to control mice (Fig 4B–4D), suggesting a type2 impairment. This was paralleled by a significantly higher rate of reduction in the number of IL-4-producing CD4^+^ T cells in *S*. *mansoni*-infected i^Cre-/+^IL-4Rα^-/lox^ Tam2 animals (~50%, S4C Fig) when compared to the minimal reduction in IFNγ-producing CD4^+^ T cells reported (~20%, S4D Fig). This impaired IL-4 production was confirmed within the supernatant of anti-CD3-stimulated MLN cells from *S*. *mansoni*-infected i^Cre-/+^IL-4Rα^-/lox^ Tam2 animals by ELISA where a greatly diminished production of other type-2 cytokines as well, i.e. IL-13, IL-5 and IL-10, amid rather minimally altered IFNγ responses (Fig 4E) was observed. Subsequently, Type 2 antibody responses (IgG1 and total IgE) appeared markedly reduced, whereas Type 1 antibodies (IgG2a) were similar to control mice (Fig 4F–4H). However, liver egg burden was similar between the different groups (Fig 4I), ruling out a differential level of infection as the cause of the observed diminished type-2 responses in i^Cre-/+^IL-4Rα^-/lox^ Tam2 and IL-4Rα^-/-^ mice. Together, these results demonstrate that knocking down IL-4Rα at the early acute phase of experimental schistosomiasis considerably diminishes host ability to subsequently mount a type-2 immune response. Liver granuloma size (Fig 4J and 4K) and fibrosis (Fig 4L and 4M) were reduced in *S*. *mansoni*-infected i^Cre-/+^IL-4Rα^-/lox^ Tam2 similar to global IL-4Rα^-/-^ mice, translating into a significantly reduced level of hepato- (Fig 4N) and splenomegaly (Fig 4O) compared with IL-4Rα-responsive control mice. As expected, from our previous mortality studies [25,27], body weights of *S*. *mansoni*-infected IL-4Rα^-/-^ and i^Cre-/+^IL-4Rα^-/lox^ Tam2 mice rapidly declined starting 6 weeks post-infection (Fig 4P) that preceded the death of these animals (Fig 4Q) when compared to IL-4Rα-responsive control mice. Bleeding was visible in the gut of the animals that rapidly succumbed to infection following removal of IL-4Rα. Taken together, these results suggest that IL-4Rα knockdown at the early acute phase of experimental schistosomiasis considerably diminishes the host ability to mount a protective fibro-granulomatous response around the *S*. *mansoni* eggs and this was associated with gut bleeding, rapid weight loss and premature death.

### IL-4Rα knockdown after egg production (week 6) exacerbates acute schistosomiasis

**Fig 5:**
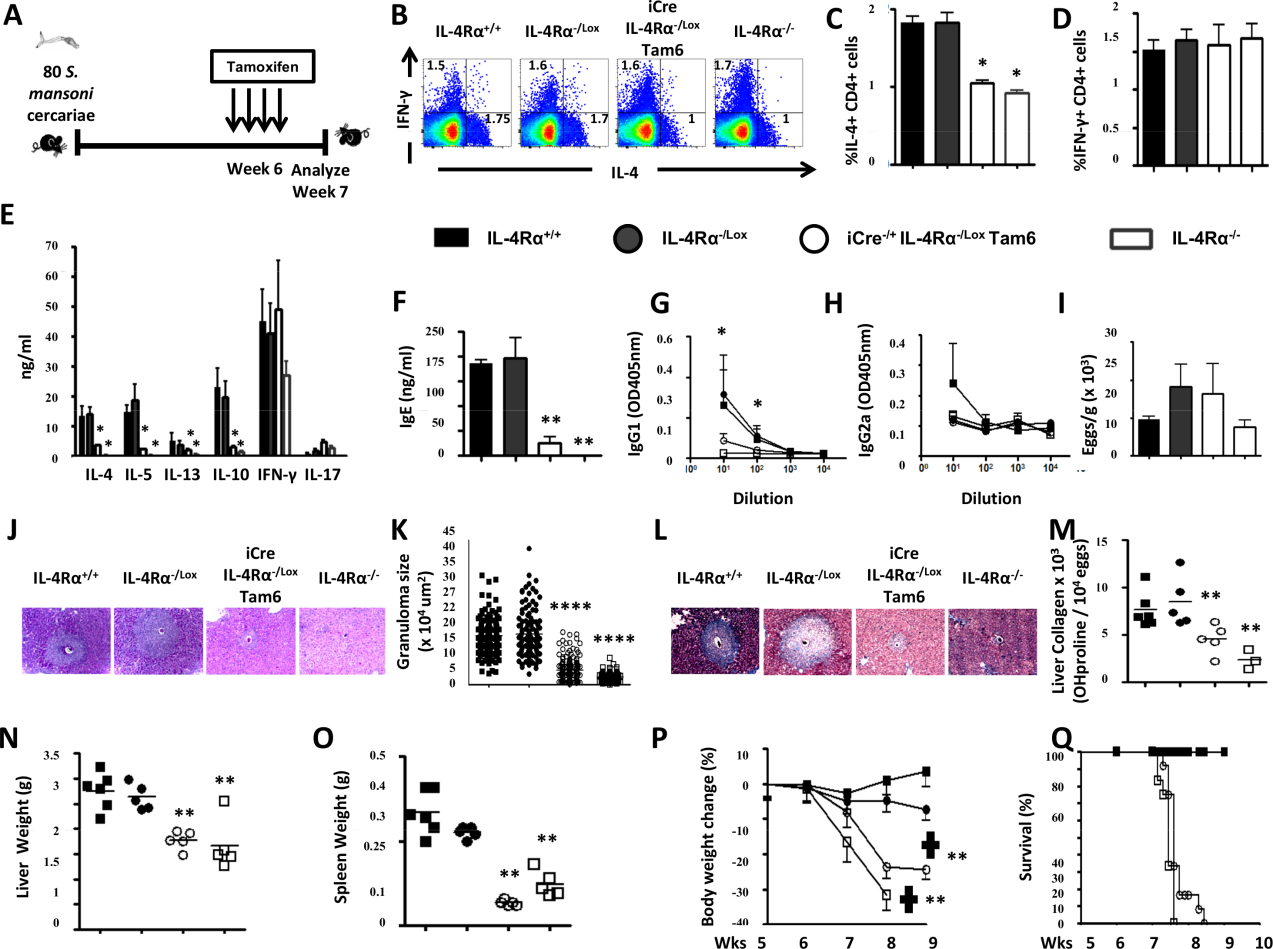
Immunological and histopathological profile of *S*. *mansoni-*infected mice after knocking down IL-4Rα 6 weeks post-infection. **A.** Experimental design. **B.** Representative plot of MLN cytokine-producing CD3^+^CD4^+^ T cells after stimulation with PMA/Ionomycin/Monensin cocktail. Summaries of IL-4-producing (**C**) and IFNγ-producing (**D**) CD3^+^CD4^+^ T cell frequencies from 2 independent experiments conducted with 5–10 mice are shown **E.** Cytokine release detected by ELISA in the supernatant of anti-CD3 stimulated MLN cells. **F.** Total seric IgE in *S*. *mansoni-infected* mice. SEA-specific seric IgG1 (**G**) and IgG2a (**H**) isotype antibodies. **I.** Liver Egg burden. **J.** Formalin-fixed Hematoxylin/Eosin-stained sections of liver tissue from infected animals for morphological analyses (displayed here at 100X). **K.** Area sizes of egg-surrounding granuloma are computed. **L.** Formalin-fixed CAB-stained sections of liver tissue from infected animals for collagen detection (displayed here at 100X). **M.** Hydroxyproline content measured by colorimetry is displayed as a measure of tissue collagen content. Liver (**N**) and Spleen (**O**) weights. **P.** Body weight change over time following *S*. *mansoni* infection. **Q.** Survival curve following *S*. *mansoni* infection. Each experiment was conducted at least twice with 5–10 mice per group. Data are expressed as mean ± SD; NS = p > 0.05; * = p < 0.05; ** = p < 0.01; *** =, p < 0.001; **** = p < 0.0001.

As impaired viability of *S*. *mansoni*-infected mice following IL-4Rα knockdown at 6 weeks post-infection (Tam6) was observed (Fig 3B), the associated immune and histopathological responses of Tam6-fed RosaCreER^T2-/+^IL-4Rα^-/lox^ mice (termed i^Cre-/+^IL-4Rα^-/lox^ Tam6, Fig 5A) was investigated. As expected, surface IL-4Rα protein on lymphocytes from *S*. *mansoni*-infected i^Cre-/+^IL-4Rα^-/lox^ Tam6 mice was abrogated as demonstrated by flow cytometry (S5A Fig and S5B Fig). A significant reduction of T lymphocytes in the MLN of *S*. *mansoni*-infected i^Cre-/+^IL-4Rα^-/lox^ Tam6 animals at week 7 post infection (S5C Fig and S5D Fig) was observed. *Ex vivo* stimulation and subsequent intracellular FACS analysis of MLN cells from *S*. *mansoni*-infected i^Cre-/+^IL-4Rα^-/lox^ Tam6 animals at week 7 post infection revealed impaired IL-4 production, similar to global IL-4Rα^-/-^ mice (Fig 5B and 5C), whereas IFN-γ responses were similar compared to IL-4Rα^-/lox^ and IL-4Rα^+/+^ control mice (Fig 5B and 5D). This suggests an impairment of type 2 immune responses in *S*. *mansoni*-infected i^Cre-/+^IL-4Rα^-/lox^ Tam6 animals as confirmed by the drastic reduction of IL-4-producing CD4^+^ T cell numbers in the MLN (~83%, S5E Fig), that paralleled a significant but less important reduction of IFNγ-producing CD4^+^ T cell numbers (~60%, S5F Fig). This reduction of IL-4 production was confirmed by anti-CD3-stimulated MLN cells from *S*. *mansoni*-infected i^Cre-/+^IL-4Rα^-/lox^ Tam6 mice and analysis of the released cytokines by ELISA (Fig 5E). Consistently, we observed a significant decrease in the production of type 2 cytokines, i.e. IL-4 and IL-10, but minimally altered IFN-γ responses (Fig 5E). As a result of reduced IL-4, type 2 antibody responses (IgG1 and total IgE) were markedly reduced (Fig 5F and 5G), whereas type 1 antibodies (IgG2a) were similar to control mice (Fig 5H). Liver egg burden was similar between the different groups (Fig 5I), ruling out a differential level of infection as the cause of the observed diminished type 2 responses in i^Cre-/+^IL-4Rα^-/lox^ Tam6 and IL-4Rα^-/-^ mice. Together, these results demonstrate that knock down of IL-4Rα after egg deposition does diminish host ability to maintain the type 2 immune responses. Reduced type 2 responses decreases pathological features, including liver granuloma size (Fig 5J and 5K) and fibrosis (Fig 5L and 5M), hepato- (Fig 5N) and splenomegaly (Fig 5O) compared with IL-4Rα-responsive control mice. However, the body weights of *S*. *mansoni*-infected i^Cre-/+^IL-4Rα^-/lox^ Tam6 mice rapidly declined following Tamoxifen-driven removal of IL-4Rα at 6 weeks post-infection similar to IL-4Rα^-/-^ mice (Fig 5P) and culminated into the early death of these animals (Fig 5Q), when compared to IL-4Rα-responsive control mice. Bleeding was visible in the gut of the animals that rapidly succumbed to infection following removal of IL-4Rα. No premature mortality was reported with *S*. *mansoni*-infected Tam6-fed IL-4Rα^+/+^ (control for Tamoxifen side effects), RosaCreER^T2-/+^IL-4Rα^+/+^ (control for activated CreER^T2^) and RosaCreER^T2-/+^IL-4Rα^-/lox^ (control for CreER^T2^ Tamoxifen-independent activity) mice when compared to *S*. *mansoni*-infected IL-4Rα^+/+^ (positive control) mice (S6 Fig) ruling out any non-specific effect(s) of Tamoxifen or CreERT2 as mediator(s) of the impaired survival of *S*. *mansoni*-infected i^Cre-/+^IL-4Rα^-/lox^ Tam6 mice. Taken together, these results show that IL-4Rα knockdown after egg deposition during the acute phase of experimental schistosomiasis considerably diminishes the host ability to maintain a type 2 immune response around the *S*. *mansoni* eggs which associates with gut bleeding, rapid weight loss and premature death.

### IL-4Rα knockdown during chronic schistosomiasis (16 weeks) ameliorates disease

**Fig 6:**
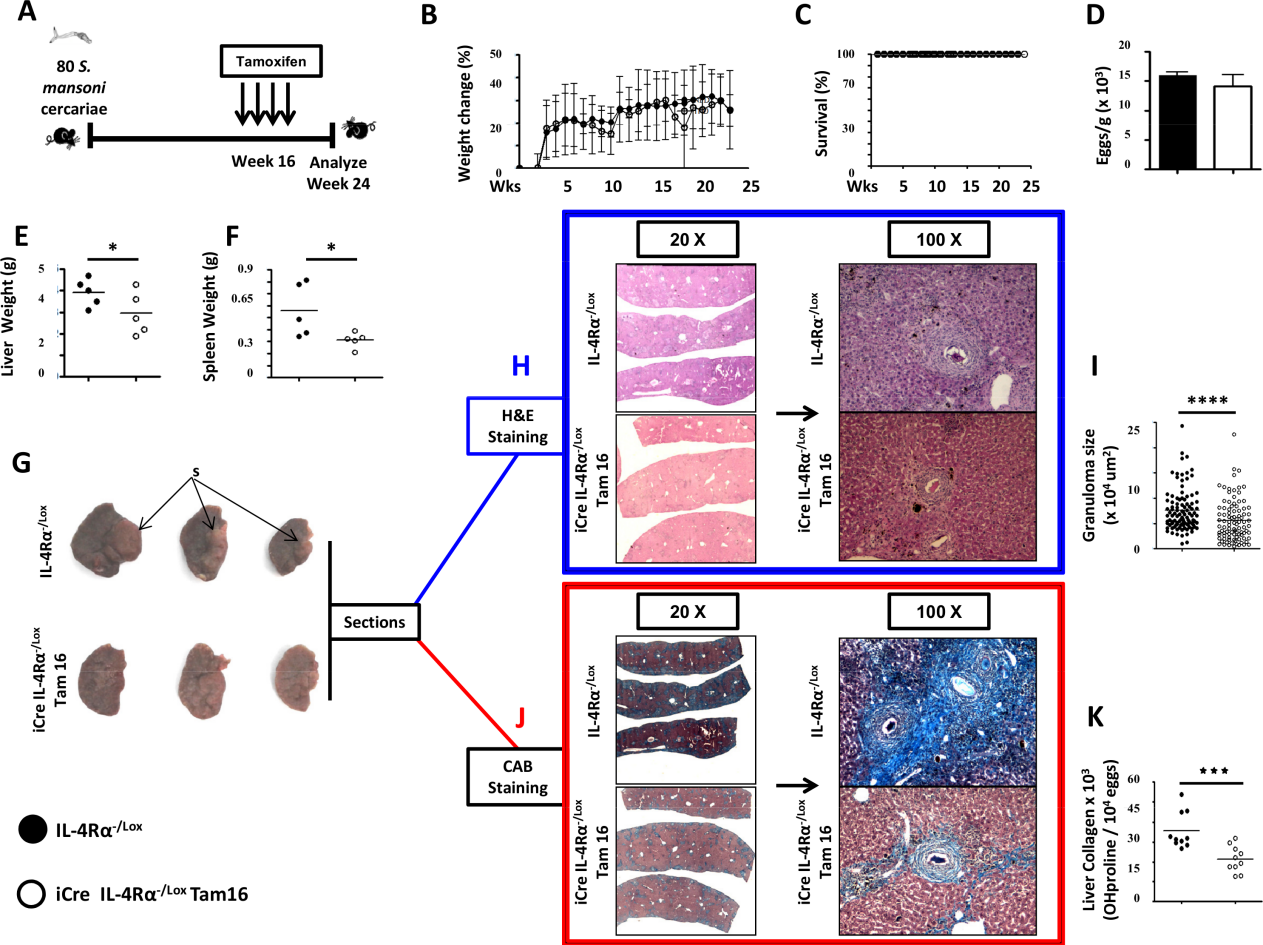
Histopathological profile of *S*. *mansoni-*infected mice after knocking down IL-4Rα 16 weeks post-infection. **A.** Experimental design. **B.** Body weight change over time following *S*. *mansoni* infection. **C.** Survival curve over 24 weeks following *S*. *mansoni* infection and IL-4Rα knockdown 16 weeks post-infection. **D.** Liver egg burden. **E.** Liver weights. **F.** Spleen weights. **G.** Representative photographs of liver lobes from mice infected for 24 weeks with *S*. *manson*i with and without IL-Rα signaling interruption at week 16. Note the formed acellular scar-like structure (S) in the livers of control mice. **H.** Formalin-fixed sections of the liver lobes were performed and stained with Hematoxylin/Eosin (H&E). **I.** Area size of egg-surrounding granuloma computed from H&E stained sections. **J.** Formalin-fixed sections of the liver lobes were stained with CAB for collagen detection (in Blue). (**K)** Hydroxyproline content measured by colorimetry is displayed as a measure of tissue collagen content. Each experiment was conducted at least twice with 5–10 mice per group. Data are expressed as mean ± SD; NS = p > 0.05; * = p < 0.05; ** = p < 0.01; *** =, p < 0.001; **** = p < 0.0001.

*S*. *mansoni*-infected mice following IL-4Rα knockdown at 16 weeks post-infection, i.e. i^Cre-/+^IL-4Rα^-/lox^ Tam16 mice (Fig 6A) did not result in any weight loss (Fig 6B) or mortality (Fig 6C), for up to 24 weeks post infection. Liver egg burden was similar between the control IL-4Rα^-/lox^ (Fig 6D), ruling out a differential level of infection between both groups of mice. IL-4Rα knockdown considerably reduced liver (Fig 6E) and spleen (Fig 6F) enlargement in chronically infected mice. Apparent scarification was visible on the liver lobes of control mice whereas IL-4Rα knockdown resulted in the removal/reversal/inhibition of liver scarification (Fig 6G). Moreover, IL-4Rα knockdown considerably reduced granuloma size (Fig 6H and 6I) and collagen levels (Fig 6J and 6K) in the livers of chronically infected mice. These data indicated that IL-4Rα knockdown ameliorate granulomatous inflammation, hepato- and splenomegaly and liver fibrosis during chronic schistosomiasis further consolidating the idea of a deleterious role for IL-4Rα signaling in mediating fibroproliferative pathology during chronic schistosomiasis.

**Fig 7:**
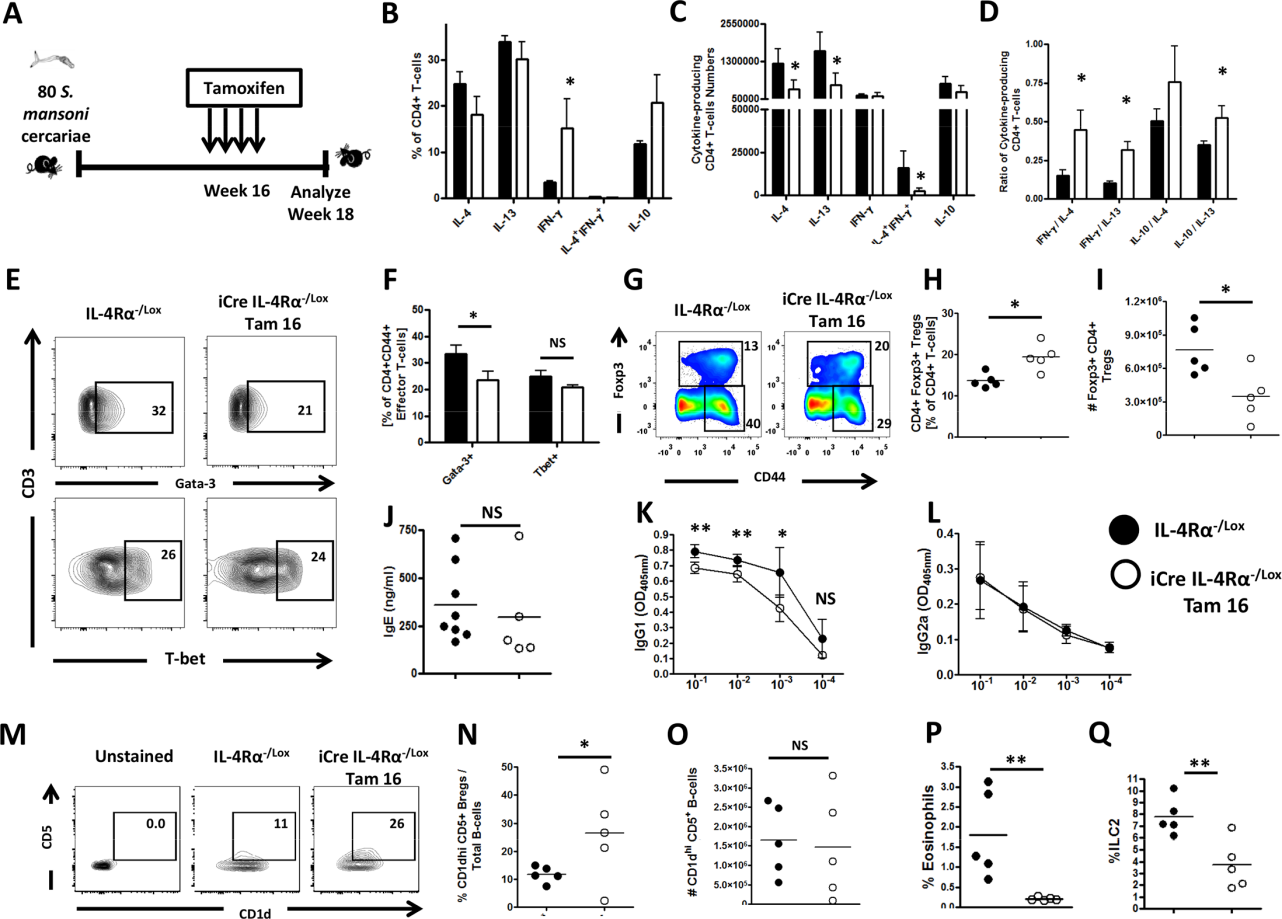
Immunological polarization of *S*. *mansoni-*infected mice after knocking down IL-4Rα 16 weeks post-infection. **A.** Experimental design for Immunological assays. Percentages (**B**), numbers (**C**) and ratios (**D**) of cytokine-producing CD4^+^ T cells after stimulation with PMA/Ionomycin/Monensin cocktail. **E.** Percentages of various transcription factor-expressing CD4^+^ T cells (*ex-vivo*) summarized in **F. G.** Representative plot of MLN Foxp3^+^CD4^+^ regulatory T cells (gated on CD3^+^CD4^+^ Lymphocytes). Summaries of frequencies (**H**) and total cell numbers (**I**) of MLN Foxp3^+^ Tregs. **J.** Total seric IgE in *S*. *mansoni-infected* mice. SEA-specific seric IgG1 (**K**) and IgG2a (**L**) isotype antibodies. **M.** Representative plot of MLN CD1d^hi^CD5^+^ regulatory B cells (gated on CD19^+^ Lymphocytes). Summaries of frequencies (**N**) and total cell numbers (**O**) of CD1d^hi^CD5^+^ regulatory B cells (gated on CD19^+^ Lymphocytes) per MLN. **P.** Frequencies of SSC^hi^ SiglecF^+^ eosinophils within total MLN cells. **Q.** Frequencies of MLN Lin^-^ T1/ST2^+^ ICOS^+^ (ILC2) cells. Each experiment was conducted at least twice with 5–10 mice per group. Data are expressed as mean ± SD; NS = p > 0.05; * = p < 0.05; ** = p < 0.01; *** =, p < 0.001; **** = p < 0.0001.

To analyse the immune polarization and responses that are triggered by IL-4Rα knockdown during chronic schistosomiasis and associate with the amelioration of tissue disease, IL-4Rα was knockdown in mice chronically infected with *S*. *mansoni* at week 16 post infection and the immune response analyzed at week 18 post infection (Fig 7A). A significant reduction of CD4^+^ (S7A Fig) and CD8^+^ (S7B Fig) T lymphocytes in the MLN of *S*. *mansoni*-infected i^Cre-/+^IL-4Rα^-/lox^ Tam16 animals at week 18 post infection was observed. Our analyses revealed a reduced Th2-mediated production of IL-4 and IL-13, but present IFN-γ and IL-10 production by MLN T cells of IL-4Rα knockdown animals, as judged by frequencies (Fig 7B, gated as per S7C Fig), total numbers (Fig 7C) and ratios (Fig 7D) of cytokine-producing MLN CD4^+^ T cells (S6 Fig and Fig 7B). Canonical transcription factor analysis (Fig 7E) confirmed this conclusion with reduction of GATA3 but normal Tbet production in effector T cells (Fig 7E and 7F). Interestingly, the frequencies of Foxp3^+^ regulatory T cell responses were increased in the MLNs of *S*. *mansoni*-infected i^Cre-/+^IL-4Rα^-/lox^ Tam16 mice (Fig 7G and 7H), when compared with *S*. *mansoni*-infected IL-4Rα^-/lox^ control mice. However, most likely as a result of total CD4^+^ T cell drop (S7A Fig), Treg cell numbers were reduced following Tam16 treatment in *S*. *mansoni*-infected i^Cre-/+^IL-4Rα^-/lox^ Tam16 animals when compared to their littermate controls (Fig 7I). Serum titers of type 2 antibodies (IgG1 and total IgE) were reduced (Fig 7J and 7K) but not type 1 antibodies (IgG2a, Fig 7L), supporting reduced type 2 responses in *S*. *mansoni*-infected i^Cre-/+^IL-4Rα^-/lox^ Tam16 mice. Of interest, regulatory B cell frequencies increased during infection and particularly in infected i^Cre-/+^IL-4Rα^-/lox^ Tam16 mice (Fig 7M and 7N) amid a rather stable total count in *S*. *mansoni*-infected i^Cre-/+^IL-4Rα^-/lox^ Tam16 animals when compared to littermate controls (Fig 7O).

Innate type 2 immune effectors i.e. eosinophils [34,35], ILC2 [36] and macrophages [37] have been positively linked to liver fibrosis, the pathophysiological process that drives the host morbidity during chronic schistosomiasis. Conversely, arginase expression by macrophages has been shown to counter tissue inflammation and fibrosis [38]. The analysis of the MLN cells of *S*. *mansoni*-infected i^Cre-/+^IL-4Rα^-/lox^ Tam16 mice for these cell types by flow cytometry (S7D Fig–S7G Fig) revealed that the pro-fibrotic innate effectors i.e. eosinophils (Fig 7P, S8A Fig and S8B Fig), ILC2 (Fig 7Q and S8C Fig) and macrophages (S8D Fig) were significantly diminished. Conversely, the mean arginase expression by macrophages (S8E Fig) was not affected in *S*. *mansoni*-infected i^Cre-/+^IL-4Rα^-/lox^ Tam16 mice, when compared to *S*. *mansoni*-infected IL-4Rα^-/lox^ control mice. This suggests that IL-4Rα knockdown in chronically infected mice does skew the MLN response away from a pro-fibrotic response. Taken together, these results suggest that knocking down IL-4Rα at the chronic phase of experimental schistosomiasis considerably skews the host immune response away from the type 2 arm of the immune response, fosters a qualitatively more regulatory, anti-inflammatory and anti-fibrotic profile with no deleterious effect on host survival.

## Discussion

Taking advantage of a newly established temporal inducible IL-4Rα deficient mouse model, we demonstrated that interrupting IL-4Rα mediated signaling prevents the onset and maintenance of egg-driven type 2 immune responses and its associated fibro-granulomatous inflammation during schistosomiasis. Whereas early knockdown of the receptor during the acute phase of the disease led to aggravated morbidity and mortality, late targeting at the chronic phase considerably ameliorated fibrogranulomatous inflammation and reduced hepato- and splenomegaly without impairing the animal viability. Amelioration of chronic schistosomiasis pathology was further associated with reduction of type 2 immune effector responses but expansion of regulatory T and B cells, suggesting that IL-4Rα mediated immune responses are detrimental in chronic schistosomiasis. Hence, therapeutic intervening of IL-4Rα mediated signaling to reduce type 2 responses might provide a strategy to ameliorate fibroproliferative pathology in diseases like chronic schistosomiasis.

The group of Cheever *et al*. (1994) were the first to show that abrogation of type 2 immune responses in *S*. *mansoni*-infected mice resulted in impaired granulomatous inflammation around the trapped eggs in tissue. A subsequent study by Chiaramonte *et al*. [21] showed the importance of IL-13-mediated signaling in fibrogenesis through the blockade of IL-13. Moreover, this group further reported a critical role for IL-13 in granuloma formation induced by *S*. *mansoni* eggs [39]. An independent group further reported on the achievement of significantly reduced tissue fibrosis by blocking type 2 responses in *S*. *mansoni*-infected mice with anti-IL-4 antibody treatment [40]. This indicated that IL-4-orchestrated type 2 responses, as well as IL-13-driven responses, are all causally linked to fibrogranulomatous pathology. More recently, using Schistosomiasis-infected IL-4Rα deficient mice, we and others demonstrated reduced fibrogranulomatous inflammation. However, these mice died during acute schistosomiasis due to cachexia [25]. Using inducible IL-4Rα deficient mice in the present study, we now dissected the role of IL-4/IL-13-mediated type 2 responses during acute and chronic murine schistosomiasis. IL-4Rα removal (knockdown) early during schistosomiasis infection led to impaired type 2 responses with reduced fibrogranulomatous inflammation around the trapped eggs of the parasites, as demonstrated before. This resulted in exacerbated morbidity and premature death of the animals, as demonstrated previously in IL-4Rα deficient mice [25]. Thus, IL-4Rα-elicited type 2 immune effector responses like granuloma formation and fibrosis are important for the host survival during acute schistosomiasis. This concept has been previously established where a tissue protective role of these responses against the toxic secretions of the parasite eggs was suggested [41]. Interestingly, liver integrity was not affected after acute knockdown of IL-4Rα in *S*. *mansoni*-infected animals. This finding argues against liver toxicity being the pathological event that drives death in these animals. A more likely explanation for their premature death would be the intensive gut bleeding reported in our previous study on IL-4Rα deficient mice [25], and similarly observed in this study. As a result of the compromised gut integrity, bacteria would translocate to the blood stream and death by septic shock would ensue, as previously demonstrated [25].

Tamoxifen-induced knockdown of the IL-4Rα after egg deposition during the chronic phase (16 weeks post-infection) uncovered a hitherto unappreciated facet of the IL-4Rα mediated type 2 responses. Indeed, IL-4Rα knockdown during chronic schistosomiasis did not lead to gut bleeding and did not affect animal viability but ameliorated liver pathology with reduced granuloma size and fibrosis in the liver and no visible scarification and reduced level of liver and spleen enlargement. This clearly suggests that IL-4Rα mediated type 2 responses are detrimental during chronic schistosomiasis and the cause for fibroproliferative liver pathology. Of interest, regulatory T and B cell compartments were significantly increased following IL-4Rα removal during chronic schistosomiasis. It is tempting to associate the beneficial effect of IL-4Rα blockade on tissue pathology during chronic schistosomiasis to the enhanced regulatory response observed. In fact, previous studies have reported an amelioration of the fibrogranulomatous inflammation during chronic schistosomiasis by Foxp3^+^ regulatory T cells [42,43]. Whether IL-4Rα mediated signaling causally dictates the anti-inflammatory and anti-fibrotic activities of these regulatory cells during chronic schistosomiasis is not known.

As of now, a role for IL-4Rα signaling in the development of immune hyporesponsiveness after chronic exposure of host immune cells to schistosomal antigens has been demonstrated [44]. This further re-emphasizes the potential of IL-4Rα in modulating the dynamics of the host regulatory responses during chronic diseases such as schistosomiasis. Such a potential has already been widely reported with a negative regulation of Foxp3^+^ Tregs and a loss of their suppressive capacity suggested to occur when the IL-4Rα signaling was solicited [45–47]. Alternatively, however, the remnant IL-4Rα mediated signaling in Tam16 mice argues against the absence of Th2 responses as the sole driver of the ameliorated pathology observed. A rather noticeable finding is the upregulation of other cytokines i.e. IL-10 and IFNγ resulting in a better balanced cytokine profile between T cells producing IL-4, IFN-γ and/or IL-10. Consequently, the impairment of IL-4Rα mediated signaling during chronic schistosomiasis by inducing a more equilibrated and mixed Th profile might prevent untoward immune polarization and tissue immunopathology. This hypothesis is strongly supported by the recently demonstrated role for immune balance rather than strong immune polarization in controlling fibrogranulomatous pathology during experimental schistosomiasis [48]. Further experiments are now required to empirically disentangle these hypotheses. What remains clear and worth focus at present is the fact that targeting IL-4Rα mediated signaling for the management of non-communicable type 2-mediated diseases in humans is in advanced clinical trials [49–51]. Understandably, building on the present study, the translatability of targeting IL-4Rα mediated signaling during fibroproliferative diseases like chronic schistosomiasis is further supported.

What do we add to the current knowledge on the control of fibroproliferative disease? It should be recalled that our present report builds on the previous observations made during IL-13 blockade experiments where a key role for this cytokine, and the indication of the potential of the IL-4Rα signaling axis in driving fibroproliferative responses during experimental schistosomiasis was defined [21]. In as much as an efficient anti-fibrotic strategy already transpired from the sole blockade of IL-13 [21], the noticeable and independent pro-fibrotic effect of IL-4 [21,40] altogether argues for the higher anti-fibrotic potential of dually targeting IL-4 and IL-13 by blocking IL-4Rα rather than IL-13 alone. The picture might not be that straightforward, however, as caution should also be exerted in dually targeting IL-4 and IL-13 via IL-4Rα given that IL-4 unlike IL-13 is critical for type 2 immune responsiveness. A state of immune deficiency might therefore arise from IL-4Rα targeting as opposed to IL-13 targeting where Th2 responses are optimally elicited [21]. Also, consistent with the observation that IL-13 targeting was not toxic for the host [21], our present report shows that IL-4R targeting does not impair animal fitness. This strongly argues for the safety of our approach. Conclusively, as of yet, one could therefore speculate on an added value of targeting IL-4Rα rather than just IL-13 given the different profibrotic potentials of IL-13 [21,39,52,53] and IL-4 [40,54–58] as both cytokines signal through IL-4Rα. Clearly, such a conclusion would still need to be experimentally validated.

In summary, we provide evidence on the role of IL-4Rα during experimental schistosomiasis whereby early signaling helps the host survive the acute phase of the disease whereas signaling at the late chronic phase mediate the morbidity. Targeting IL-4Rα might therefore represent a novel therapeutic strategy against the fibroproliferative pathology that drives the morbidity of fibrotic diseases like chronic schistosomiasis.

## Materials and methods

### Mice

IL-4Rα^-/-^, IL-4Rα^-/lox^ and CreER^T2^ mice on a C57/BL6 background were previously described [2,25,33]. We generated a novel inducible IL-4Rα deleting mouse strain (*RosaCreER*^*T2-/+*^*IL-4Rα*^*-/lox*^) by intercrossing transgenic *RosaCreER*^*T2-/+*^ mice with IL-4Rα^-/-^ and IL-4Rα^Lox/Lox^ mice. CreER^T2^ transgenic negative littermates (IL-4Rα^-/lox^) expressing functional IL-4Rα were used as controls in all experiments. Mice were maintained in the University of Cape Town specific pathogen-free animal facility in accordance with the guidelines established by the Animal Research Ethics committee of the Faculty of Health Science of the University of Cape Town and the South African Veterinary Council (SAVC).

### Ethics statement

All animal experiments were conducted under strict recommendation of the South African national guidelines and of the University of Cape Town practice for laboratory animal procedures as outlined in protocols 010/048 and 014/003 reviewed and approved by the Animals Research Ethics Committee of the Faculty of Health Science of the University of Cape Town. Both male and female mice aged 6–12 weeks were used for all experiments. Care was taken under these protocols to minimize animal suffering in accordance with the guidelines of the Animal Research Ethics committee of the Faculty of Health Science of the University of Cape Town and the South African Veterinary Council (SAVC).

### Parasite infection and Tamoxifen administration

Mice were infected percutaneously via the abdomen with 35, 80 or 100 cercariae, as indicated, with a Puerto Rican strain of *Schistosoma mansoni* obtained from infected *Biomphalaria glabrata* (a generous gift from Adrian Mountford, York, UK). Eggs were purified from digested sections of liver or ileum from infected animals and counted at 40× magnification as previously described [40]. To activate *il-4rα* gene excision by CreER^T2^, Tamoxifen (Sigma, Deisenhofen, Germany) solubilized in vegetable oil was administered by oral gavage to mice for four consecutive days (2.5mg/day).

### Genotypic characterization

Polymerase chain reaction was used to confirm the genotype of RosaCreER^T2-/+^IL-4Rα^-/lox^ mice. PCR conditions were 94°C for 2 minute, 94°C for 20 seconds, 45°C for 30 seconds, and 72°C for 20 seconds for 40 cycles. To quantify the efficiency of deletion, real-time PCR was performed on genomic DNA from liver and spleen cells using primers specific for IL-4Rα exon 5 (control) and exon 8 (deleted by CreER^T2^ activation) as described previously [25].

### Flow cytometry

Il-4Rα surface expression was detected on splenocytes, lymph node cells, lung cells, hepatocytes, bone marrow cells and peritoneal exudate cells by phycoerythrin (PE) anti-CD124 (IL-4Rα, M-1). Cell subpopulations were identified with Alexa Fluor 700, BD Horizon V500, BD Horizon V450, PerCP-Cy5.5, APC, APC-Cy7, Fluoroscein isothiocyanate, PE, PE-Cy7 or biotinylated monoclonal antibodies against CD3, CD4, CD8, CD19, Lineage, CD1d, CD5, Foxp3, Gata-3, T-bet, IL-4, IL-13, IFN-γ, IL-10, F4/80, Ly6G, CD11c, MHCII, SiglecF, T1/ST2, ICOS, Arginase, CD11b. Biotin-labeled antibodies were detected by Allophycocyanin or PercP-Cy5.5. For staining, cells (1x 10^6^) were labeled and washed in PBS, 3% FCS and 0.1% NaN_3_. Between each step of staining, cells were washed extensively. For intracellular cytokine staining, cells were restimulated with a cocktail of PMA/Ionomycin/Monensin for 4h at 37°C then fixed in 2% PFA, permeabilized and cytokine production was analyzed as previously described [25]. For intranuclear staining, a commercially available transcription buffer set (BD Bioscience) was used as per the manufacturer’s instructions. All antibodies were from BD Pharmingen (San Diego, CA) except where noted otherwise. Stained cells were then acquired on a LSR Fortessa machine (BD Immunocytometry system, San Jose, CA, USA) and data were analyzed using Flowjo software (Treestar, Ashland, OR, Usa).

### *In vitro* stimulation

#### Stimulation with cytokines

Splenocytes or MLN cells were seeded at a density of 5 x 10^6^ cells/ml and stimulated with 1 ng/ml of rIL-4 for 48h. After incubation, the cells were harvested and washed in PBS, 3% FCS and 0.1% NaN_3_ before labeling of IL-4Rα for flow cytometry. To avoid nonspecific binding, cells were incubated with a mixture containing rat sera and unlabeled anti-CD32/CD16 Antibody (2.4G2). In another series of stimulation, cultures incubated for 48h with different amounts of rIL-2 then incubated with CellTiter-blue assay reagent (Promega, Germany) for 4h before OD measurement at 570/620 to measure the cell viability.

#### *Ex vivo* restimulation of MLN

Single cell suspensions from MLN cells of *S*. *mansoni*-infected animals were prepared by pressing the MLN through 70μm cell-strainers. Cells were resuspended in complete IMDM (Gibco) supplemented with 10% FCS (Gibco) and Penicillin and Streptomycin (100 U/ml and 100 μg/ml, Gibco). The cells were cultured at 1×10^6^ cells/ml in 96-well plates coated with α-CD3 (20 μg/ml) or supplemented with SEA (20 μg/ml) and incubated at 37°C in a humidified atmosphere containing 5% CO_2_. Supernatants were collected after 72 h and cytokines were measured by sandwich ELISA as previously described [33].

### Serum analyses

#### Antibody titers

*S*. *mansoni* antigen-specific serum antibody isotypes and total IgE titers from infected mice were determined as follows. Blood was collected in serum separator tubes (BD Bioscience, San Diego, CA) and centrifuged at 8 000×g for 10 min at 4°C to separate serum. The flat-bottom 96-well plates were coated with 10 μg/ml SEA, blocked with 2% (w/v) milk powder for 2 h at 37°C and samples were loaded and incubated overnight at 4°C. Alkaline phosphatase labeled secondary antibody was added and incubated for 2 h at 37°C. The plates were developed by addition of 4-nitrophenyl substrate (Sigma). The absorbance was read at 405 nm using VersaMax microplate spectrophotometer (Molecular Devices, Germany).

#### Liver enzymes

Hepatocellular damage was assessed by measuring the serum levels of alanine transaminase at the National Health Laboratory Service of South Africa (Cape Town).

### Histology and hydroxyproline quantification

Tissue samples were fixed in neutral buffered formalin, processed, and 5–7 μm sections stained with hematoxylin and eosin (H & E). Granuloma diameter of 20–50 granulomas per animal was determined using an ocular micrometer (Nikon NIS-Elements, Nikon Corporation, Tokyo, Japan). For fibrosis assessment, tissue sections were stained with chromotrope 2R and analine blue solution (CAB) and counterstained with Wegert's hematoxylin for collagen staining. Complementarily, a modified protocol of tissue hydroxyproline quantification was used [59]. In brief, weighed liver samples were hydrolyzed and the supernatant was neutralized with 1% phenolphthalein and titrated against 10 M NaOH. An aliquot was mixed with isopropanol and added to a chloramine-T/citrate buffer solution (pH 6.0) (Sigma). Ehrlich's reagent solution was added and measured at 570 nm. Hydroxyproline levels were calculated by using 4-hydroxy-L-proline (Calbiochem) as standard, and results were expressed as μg hydroxyproline per weight of liver tissue that contained 10^4^ eggs.

### Statistics

Statistical analysis was conducted using GraphPad Prism 4 software (http://www.prism-software.com). Data were calculated as mean ± SD. Statistical significance was determined using the unpaired Student's t test, One-Way or Two-Way ANOVA with Bonferroni's post test, defining differences to C57BL/6, IL-4Rα^-/lox^ or oil-treated RosaCreER^T2-/+^IL-4Rα^-/lox^ as significant (*, p≤0.05; **, p≤0.01; ***, p≤0.001).

## Supporting information

S1 Fig Unaltered cellularity, cellular and humoral responses at baseline in naive iCre^-/+^ IL-4Rα^-/lox^ mice. **A.** Experimental design. **B.** Spleen weights. **C.** Total liver cell numbers. **D.** Hepatocellular damage at baseline. Alanine transaminase (ALT) sera concentration. **E.** Total seric IgE at baseline. F. Unimpaired IL-2-mediated proliferation of splenocytes in the absence of IL-4Rα. Splenocytes from control (IL-4Rα^-/lox)^, Tamoxifen-treated iCre^-/+^ IL-4Rα^-/lox^ mice and IL-4Rα^-/-^ mice were stimulated with IL-2. Metabolic activity was measured by colorimetric detection of resazurin reduction into resofurin. Mean ± SD of triplicate cultures; NS = p > 0.05; * = p < 0.05; ** = p < 0.01; *** =, p < 0.001; **** = p < 0.0001.
(TIF)

S2 Fig Spleen and MLN cellularity in naive mice. **A.** Gating strategy and average frequencies of CD3^+^ T cells, CD3^-^CD19^+^ B cells, F4/80^+^ macrophages and CD11c^+^MHCII^+^ dendritic cells in the spleen of wt (IL-4Rα^+/+^), littermate controls (IL-4Rα^-/lox^), Tamoxifen-fed iCre^-/+^IL-4Rα^-/lox^ and IL-4Rα-deficient (IL-4Rα^-/-^) mice. **B.** Frequencies of CD3^+^ T cells, CD3^-^CD19^+^ B cells, in the MLN of wt (IL-4Rα^+/+^), littermate controls (IL-4Rα^-/lox^), Tamoxifen-fed iCre^-/+^IL-4Rα^-/lox^ and IL-4Rα-deficient (IL-4Rα^-/-^) mice. Scatter plots are representative of analyses performed at least twice with 3–4 mice per group. Total CD4^+^ (**C**) and CD8^+^ (**D**) T cell numbers in the MLN of naive iCre^-/+^ IL-4Rα^-/lox^ mice 5 days following treatment.
(TIF)

S3 Fig Hepatocellular damage after *S*. *mansoni* infection and IL-4Rα knockdown. Alanine transaminase (ALT) sera concentration 18 weeks post-infection with *S*. *mansoni*. Experiment was conducted twice with 5–10 mice per group. Data are expressed as mean ± SD; NS = p > 0.05; * = p < 0.05.
(TIF)

S4 Fig Total and cytokine-producing T cells, related to Tam2 scheme. **A.** Total MLN CD4^+^ T cell numbers. **B.** Total MLN CD8^+^ T cell numbers. Total IL-4-producing (**C**) and IFNγ-producing (**D**) MLN CD4^+^ T cell numbers. Each experiment was conducted at least twice with 5–10 mice per group. Data are expressed as mean ± SD; NS = p > 0.05; * = p < 0.05; ** = p < 0.01; *** =, p < 0.001; **** = p < 0.0001.
(TIF)

S5 Fig IL-4Rα expression, whole and cytokine-producing cell counts, related to Tam6 scheme. IL-4Rα GMFI on CD3^+^CD4^+^ **(A)** T cells and CD3^-^CD19^+^ B cells **(B)** from MLN of naïve (collected 5 days following Tamoxifen treatment) vs. MLN of Tam6 *S*. *mansoni*-infected mice. **C**. Total Tam6 MLN CD4^+^ T cell numbers. **D.** Total Tam6 MLN CD8^+^ T cell numbers. Total IL-4-producing (**E**) and IFNγ-producing (**F**) MLN CD4^+^ T cell numbers under the Tam6 scheme. Each experiment was conducted at least twice with 5–10 mice per group. Data are expressed as mean ± SD; NS = p > 0.05; * = p < 0.05; ** = p < 0.01; *** =, p < 0.001.
(TIF)

S6 Fig Survival curve. Mice (10 per group) were infected percutaneously with 80 *S*. *mansoni* cercariae and monitored over time. Presented are the weekly percentages of survivors.
(TIF)

S7 Fig Gating strategies, related to the Tam16 scheme. **A.** Total Tam16 MLN CD4^+^ T cell numbers. **B.** Total Tam16 MLN CD8^+^ T cell numbers. **C.** Gating of cytokine-producing CD4^+^ T cells. **D.** Gating within MLN lymphoid and myeloid cells to define Ssc^hi^ SiglecF^+^ eosinophils (**E**), Lin^-^T1/ST2^+^ICOS^+^ ILC2 (**F**) and Fsc^hi^ F4/80^+^ macrophages,(**G**).
(TIF)

S8 Fig **Changes in total numbers of MLN cells (A), Eosinophils (B), ILC2 (C), macrophages (D) and Arginase expression by macrophages (E) in *S*. *mansoni*-infected mice following IL-4Rα knockdown at 16 weeks post-infection.** Each experiment was conducted at least twice with 3–6 mice per group. Data are expressed as mean ± SD; NS = p > 0.05; * = p < 0.05; ** = p < 0.01; *** =, p < 0.001; **** = p < 0.0001.
(TIF)

## References

[1] Colley DG, Bustinduy AL, Secor WE, King CH (2014) Human schistosomiasis. Lancet 383: 2253–2264. doi: 10.1016/S0140-6736(13)61949-2 24698483 10.1016/S0140-6736(13)61949-2 PMC4672382

[2] Ndlovu H, Brombacher F (2014) Role of IL-4Ralpha during acute schistosomiasis in mice. Parasite Immunol 36: 421–427. doi: 10.1111/pim.12080 24127774 10.1111/pim.12080 PMC4286023

[3] Pearce EJ, MacDonald AS (2002) The immunobiology of schistosomiasis. Nat Rev Immunol 2: 499–511. doi: 10.1038/nri843 12094224 10.1038/nri843

[4] Vendelova E, Camargo de LJ, Lorenzatto KR, Monteiro KM, Mueller T, Veepaschit J, Grimm C, Brehm K, Hrckova G, Lutz MB, Ferreira HB, Nono JK (2016) Proteomic Analysis of Excretory-Secretory Products of Mesocestoides corti Metacestodes Reveals Potential Suppressors of Dendritic Cell Functions. PLoS Negl Trop Dis 10: e0005061 doi: 10.1371/journal.pntd.0005061 27736880 10.1371/journal.pntd.0005061 PMC5063416

[5] Nono JK, Pletinckx K, Lutz MB, Brehm K (2012) Excretory/secretory-products of Echinococcus multilocularis larvae induce apoptosis and tolerogenic properties in dendritic cells in vitro. PLoS Negl Trop Dis 6: e1516 doi: 10.1371/journal.pntd.0001516 22363826 10.1371/journal.pntd.0001516 PMC3283565

[6] Nono JK, Ndlovu H, Abdel AN, Mpotje T, Hlaka L, Brombacher F (2017) Interleukin-4 receptor alpha is still required after Th2 polarization for the maintenance and the recall of protective immunity to Nematode infection. PLoS Negl Trop Dis 11: e0005675 doi: 10.1371/journal.pntd.0005675 28651009 10.1371/journal.pntd.0005675 PMC5501681

[7] Fallon PG, Mangan NE (2007) Suppression of TH2-type allergic reactions by helminth infection. Nat Rev Immunol 7: 220–230. doi: 10.1038/nri2039 17318233 10.1038/nri2039

[8] Wilson MS, Mentink-Kane MM, Pesce JT, Ramalingam TR, Thompson R, Wynn TA (2007) Immunopathology of schistosomiasis. Immunol Cell Biol 85: 148–154. doi: 10.1038/sj.icb.7100014 17160074 10.1038/sj.icb.7100014 PMC3437548

[9] Byram JE, von LF (1977) Altered schistosome granuloma formation in nude mice. Am J Trop Med Hyg 26: 944–956. 303056 10.4269/ajtmh.1977.26.944

[10] Buchanan RD, Fine DP, Colley DG (1973) Schistosoma mansoni infection in mice depleted of thymus-dependent lymphocytes. II. Pathology and altered pathogenesis. Am J Pathol 71: 207–218. 4541346 PMC1903958

[11] Byram JE, Doenhoff MJ, Musallam R, Brink LH, von LF (1979) Schistosoma mansoni infections in T-cell deprived mice, and the ameliorating effect of administering homologous chronic infection serum. II. Pathology. Am J Trop Med Hyg 28: 274–285. 313162 10.4269/ajtmh.1979.28.274

[12] Doenhoff M, Musallam R, Bain J, McGregor A (1979) Schistosoma mansoni infections in T-cell deprived mice, and the ameliorating effect of administering homologous chronic infection serum. I. Pathogenesis. Am J Trop Med Hyg 28: 260–263. 313161 10.4269/ajtmh.1979.28.260

[13] Fine DP, Buchanan RD, Colley DG (1973) Schistosoma mansoni infection in mice depleted of thymus-dependent lymphocytes. I. Eosinophilia and immunologic responses to a schistosomal egg preparation. Am J Pathol 71: 193–206. 4541345 PMC1903959

[14] Amiri P, Locksley RM, Parslow TG, Sadick M, Rector E, Ritter D, McKerrow JH (1992) Tumour necrosis factor alpha restores granulomas and induces parasite egg-laying in schistosome-infected SCID mice. Nature 356: 604–607. doi: 10.1038/356604a0 1560843 10.1038/356604a0

[15] Grzych JM, Pearce E, Cheever A, Caulada ZA, Caspar P, Heiny S, Lewis F, Sher A (1991) Egg deposition is the major stimulus for the production of Th2 cytokines in murine schistosomiasis mansoni. J Immunol 146: 1322–1327. 1825109

[16] Kaplan MH, Whitfield JR, Boros DL, Grusby MJ (1998) Th2 cells are required for the Schistosoma mansoni egg-induced granulomatous response. J Immunol 160: 1850–1856. 9469446

[17] Pearce EJ, Caspar P, Grzych JM, Lewis FA, Sher A (1991) Downregulation of Th1 cytokine production accompanies induction of Th2 responses by a parasitic helminth, Schistosoma mansoni. J Exp Med 173: 159–166. 1824635 10.1084/jem.173.1.159 PMC2118762

[18] Fallon PG (2000) Immunopathology of schistosomiasis: a cautionary tale of mice and men. Immunol Today 21: 29–35. 10637556 10.1016/s0167-5699(99)01551-0

[19] LaPorte SL, Juo ZS, Vaclavikova J, Colf LA, Qi X, Heller NM, Keegan AD, Garcia KC (2008) Molecular and structural basis of cytokine receptor pleiotropy in the interleukin-4/13 system. Cell 132: 259–272. doi: 10.1016/j.cell.2007.12.030 18243101 10.1016/j.cell.2007.12.030 PMC2265076

[20] Kuperman DA, Huang X, Koth LL, Chang GH, Dolganov GM, Zhu Z, Elias JA, Sheppard D, Erle DJ (2002) Direct effects of interleukin-13 on epithelial cells cause airway hyperreactivity and mucus overproduction in asthma. Nat Med 8: 885–889. doi: 10.1038/nm734 12091879 10.1038/nm734

[21] Chiaramonte MG, Donaldson DD, Cheever AW, Wynn TA (1999) An IL-13 inhibitor blocks the development of hepatic fibrosis during a T-helper type 2-dominated inflammatory response. J Clin Invest 104: 777–785. doi: 10.1172/JCI7325 10491413 10.1172/JCI7325 PMC408441

[22] Kaviratne M, Hesse M, Leusink M, Cheever AW, Davies SJ, McKerrow JH, Wakefield LM, Letterio JJ, Wynn TA (2004) IL-13 activates a mechanism of tissue fibrosis that is completely TGF-beta independent. J Immunol 173: 4020–4029. 15356151 10.4049/jimmunol.173.6.4020

[23] Brunet LR, Finkelman FD, Cheever AW, Kopf MA, Pearce EJ (1997) IL-4 protects against TNF-alpha-mediated cachexia and death during acute schistosomiasis. J Immunol 159: 777–785. 9218595

[24] Fallon PG, Richardson EJ, McKenzie GJ, McKenzie AN (2000) Schistosome infection of transgenic mice defines distinct and contrasting pathogenic roles for IL-4 and IL-13: IL-13 is a profibrotic agent. J Immunol 164: 2585–2591. 10679097 10.4049/jimmunol.164.5.2585

[25] Herbert DR, Holscher C, Mohrs M, Arendse B, Schwegmann A, Radwanska M, Leeto M, Kirsch R, Hall P, Mossmann H, Claussen B, Forster I, Brombacher F (2004) Alternative macrophage activation is essential for survival during schistosomiasis and downmodulates T helper 1 responses and immunopathology. Immunity 20: 623–635. 15142530 10.1016/s1074-7613(04)00107-4

[26] Jankovic D, Kullberg MC, Noben-Trauth N, Caspar P, Ward JM, Cheever AW, Paul WE, Sher A (1999) Schistosome-infected IL-4 receptor knockout (KO) mice, in contrast to IL-4 KO mice, fail to develop granulomatous pathology while maintaining the same lymphokine expression profile. J Immunol 163: 337–342. 10384133

[27] Dewals B, Hoving JC, Leeto M, Marillier RG, Govender U, Cutler AJ, Horsnell WG, Brombacher F (2009) IL-4Ralpha responsiveness of non-CD4 T cells contributes to resistance in schistosoma mansoni infection in pan-T cell-specific IL-4Ralpha-deficient mice. Am J Pathol 175: 706–716. doi: 10.2353/ajpath.2009.090137 19628763 10.2353/ajpath.2009.090137 PMC2716945

[28] Marillier RG, Brombacher TM, Dewals B, Leeto M, Barkhuizen M, Govender D, Kellaway L, Horsnell WG, Brombacher F (2010) IL-4R{alpha}-responsive smooth muscle cells increase intestinal hypercontractility and contribute to resistance during acute Schistosomiasis. Am J Physiol Gastrointest Liver Physiol 298: G943–G951. doi: 10.1152/ajpgi.00321.2009 20360135 10.1152/ajpgi.00321.2009

[29] Ramalingam TR, Pesce JT, Sheikh F, Cheever AW, Mentink-Kane MM, Wilson MS, Stevens S, Valenzuela DM, Murphy AJ, Yancopoulos GD, Urban JF Jr., Donnelly RP, Wynn TA (2008) Unique functions of the type II interleukin 4 receptor identified in mice lacking the interleukin 13 receptor alpha1 chain. Nat Immunol 9: 25–33. doi: 10.1038/ni1544 18066066 10.1038/ni1544 PMC2692551

[30] Vannella KM, Barron L, Borthwick LA, Kindrachuk KN, Narasimhan PB, Hart KM, Thompson RW, White S, Cheever AW, Ramalingam TR, Wynn TA (2014) Incomplete deletion of IL-4Ralpha by LysM(Cre) reveals distinct subsets of M2 macrophages controlling inflammation and fibrosis in chronic schistosomiasis. PLoS Pathog 10: e1004372 doi: 10.1371/journal.ppat.1004372 25211233 10.1371/journal.ppat.1004372 PMC4161449

[31] Nguyen KD, Qiu Y, Cui X, Goh YP, Mwangi J, David T, Mukundan L, Brombacher F, Locksley RM, Chawla A (2011) Alternatively activated macrophages produce catecholamines to sustain adaptive thermogenesis. Nature 480: 104–108. doi: 10.1038/nature10653 22101429 10.1038/nature10653 PMC3371761

[32] Feil R, Wagner J, Metzger D, Chambon P (1997) Regulation of Cre recombinase activity by mutated estrogen receptor ligand-binding domains. Biochem Biophys Res Commun 237: 752–757. doi: 10.1006/bbrc.1997.7124 9299439 10.1006/bbrc.1997.7124

[33] Mohrs M, Ledermann B, Kohler G, Dorfmuller A, Gessner A, Brombacher F (1999) Differences between IL-4- and IL-4 receptor alpha-deficient mice in chronic leishmaniasis reveal a protective role for IL-13 receptor signaling. J Immunol 162: 7302–7308. 10358179

[34] Reiman RM, Thompson RW, Feng CG, Hari D, Knight R, Cheever AW, Rosenberg HF, Wynn TA (2006) Interleukin-5 (IL-5) augments the progression of liver fibrosis by regulating IL-13 activity. Infect Immun 74: 1471–1479. doi: 10.1128/IAI.74.3.1471-1479.2006 16495517 10.1128/IAI.74.3.1471-1479.2006 PMC1418671

[35] Shen ZJ, Esnault S, Rosenthal LA, Szakaly RJ, Sorkness RL, Westmark PR, Sandor M, Malter JS (2008) Pin1 regulates TGF-beta1 production by activated human and murine eosinophils and contributes to allergic lung fibrosis. J Clin Invest 118: 479–490. doi: 10.1172/JCI32789 18188456 10.1172/JCI32789 PMC2176187

[36] McHedlidze T, Waldner M, Zopf S, Walker J, Rankin AL, Schuchmann M, Voehringer D, McKenzie AN, Neurath MF, Pflanz S, Wirtz S (2013) Interleukin-33-dependent innate lymphoid cells mediate hepatic fibrosis. Immunity 39: 357–371. doi: 10.1016/j.immuni.2013.07.018 23954132 10.1016/j.immuni.2013.07.018 PMC4172965

[37] Pellicoro A, Ramachandran P, Iredale JP, Fallowfield JA (2014) Liver fibrosis and repair: immune regulation of wound healing in a solid organ. Nat Rev Immunol 14: 181–194. doi: 10.1038/nri3623 24566915 10.1038/nri3623

[38] Pesce JT, Ramalingam TR, Mentink-Kane MM, Wilson MS, El Kasmi KC, Smith AM, Thompson RW, Cheever AW, Murray PJ, Wynn TA (2009) Arginase-1-expressing macrophages suppress Th2 cytokine-driven inflammation and fibrosis. PLoS Pathog 5: e1000371 doi: 10.1371/journal.ppat.1000371 19360123 10.1371/journal.ppat.1000371 PMC2660425

[39] Chiaramonte MG, Schopf LR, Neben TY, Cheever AW, Donaldson DD, Wynn TA (1999) IL-13 is a key regulatory cytokine for Th2 cell-mediated pulmonary granuloma formation and IgE responses induced by Schistosoma mansoni eggs. J Immunol 162: 920–930. 9916716

[40] Cheever AW, Williams ME, Wynn TA, Finkelman FD, Seder RA, Cox TM, Hieny S, Caspar P, Sher A (1994) Anti-IL-4 treatment of Schistosoma mansoni-infected mice inhibits development of T cells and non-B, non-T cells expressing Th2 cytokines while decreasing egg-induced hepatic fibrosis. J Immunol 153: 753–759. 8021510

[41] Hams E, Aviello G, Fallon PG (2013) The schistosoma granuloma: friend or foe? Front Immunol 4: 89 doi: 10.3389/fimmu.2013.00089 23596444 10.3389/fimmu.2013.00089 PMC3625856

[42] Layland LE, Rad R, Wagner H, da Costa CU (2007) Immunopathology in schistosomiasis is controlled by antigen-specific regulatory T cells primed in the presence of TLR2. Eur J Immunol 37: 2174–2184. doi: 10.1002/eji.200737063 17621370 10.1002/eji.200737063

[43] Turner JD, Jenkins GR, Hogg KG, Aynsley SA, Paveley RA, Cook PC, Coles MC, Mountford AP (2011) CD4+CD25+ regulatory cells contribute to the regulation of colonic Th2 granulomatous pathology caused by schistosome infection. PLoS Negl Trop Dis 5: e1269 doi: 10.1371/journal.pntd.0001269 21858239 10.1371/journal.pntd.0001269 PMC3153428

[44] Cook PC, Aynsley SA, Turner JD, Jenkins GR, Van RN, Leeto M, Brombacher F, Mountford AP (2011) Multiple helminth infection of the skin causes lymphocyte hypo-responsiveness mediated by Th2 conditioning of dermal myeloid cells. PLoS Pathog 7: e1001323 doi: 10.1371/journal.ppat.1001323 21445234 10.1371/journal.ppat.1001323 PMC3060168

[45] Pelly VS, Coomes SM, Kannan Y, Gialitakis M, Entwistle LJ, Perez-Lloret J, Czieso S, Okoye IS, Ruckerl D, Allen JE, Brombacher F, Wilson MS (2017) Interleukin 4 promotes the development of ex-Foxp3 Th2 cells during immunity to intestinal helminths. J Exp Med 214: 1809–1826. doi: 10.1084/jem.20161104 28507062 10.1084/jem.20161104 PMC5460998

[46] Massoud AH, Charbonnier LM, Lopez D, Pellegrini M, Phipatanakul W, Chatila TA (2016) An asthma-associated IL4R variant exacerbates airway inflammation by promoting conversion of regulatory T cells to TH17-like cells. Nat Med 22: 1013–1022. doi: 10.1038/nm.4147 27479084 10.1038/nm.4147 PMC5014738

[47] Noval RM, Burton OT, Wise P, Charbonnier LM, Georgiev P, Oettgen HC, Rachid R, Chatila TA (2015) Regulatory T cell reprogramming toward a Th2-cell-like lineage impairs oral tolerance and promotes food allergy. Immunity 42: 512–523. doi: 10.1016/j.immuni.2015.02.004 25769611 10.1016/j.immuni.2015.02.004 PMC4366316

[48] Peine M, Rausch S, Helmstetter C, Frohlich A, Hegazy AN, Kuhl AA, Grevelding CG, Hofer T, Hartmann S, Lohning M (2013) Stable T-bet(+)GATA-3(+) Th1/Th2 hybrid cells arise in vivo, can develop directly from naive precursors, and limit immunopathologic inflammation. PLoS Biol 11: e1001633 doi: 10.1371/journal.pbio.1001633 23976880 10.1371/journal.pbio.1001633 PMC3747991

[49] Beck LA, Thaci D, Hamilton JD, Graham NM, Bieber T, Rocklin R, Ming JE, Ren H, Kao R, Simpson E, Ardeleanu M, Weinstein SP, Pirozzi G, Guttman-Yassky E, Suarez-Farinas M, Hager MD, Stahl N, Yancopoulos GD, Radin AR (2014) Dupilumab treatment in adults with moderate-to-severe atopic dermatitis. N Engl J Med 371: 130–139. doi: 10.1056/NEJMoa1314768 25006719 10.1056/NEJMoa1314768

[50] Wenzel SE, Wang L, Pirozzi G (2013) Dupilumab in persistent asthma. N Engl J Med 369: 1276 doi: 10.1056/NEJMc1309809 24066755 10.1056/NEJMc1309809

[51] Wenzel S, Ford L, Pearlman D, Spector S, Sher L, Skobieranda F, Wang L, Kirkesseli S, Rocklin R, Bock B, Hamilton J, Ming JE, Radin A, Stahl N, Yancopoulos GD, Graham N, Pirozzi G (2013) Dupilumab in persistent asthma with elevated eosinophil levels. N Engl J Med 368: 2455–2466. doi: 10.1056/NEJMoa1304048 23688323 10.1056/NEJMoa1304048

[52] Chiaramonte MG, Cheever AW, Malley JD, Donaldson DD, Wynn TA (2001) Studies of murine schistosomiasis reveal interleukin-13 blockade as a treatment for established and progressive liver fibrosis. Hepatology 34: 273–282. doi: 10.1053/jhep.2001.26376 11481612 10.1053/jhep.2001.26376

[53] Chiaramonte MG, Mentink-Kane M, Jacobson BA, Cheever AW, Whitters MJ, Goad ME, Wong A, Collins M, Donaldson DD, Grusby MJ, Wynn TA (2003) Regulation and function of the interleukin 13 receptor alpha 2 during a T helper cell type 2-dominant immune response. J Exp Med 197: 687–701. doi: 10.1084/jem.20020903 12642601 10.1084/jem.20020903 PMC2193852

[54] de Jesus AR, Magalhaes A, Miranda DG, Miranda RG, Araujo MI, de Jesus AA, Silva A, Santana LB, Pearce E, Carvalho EM (2004) Association of type 2 cytokines with hepatic fibrosis in human Schistosoma mansoni infection. Infect Immun 72: 3391–3397. doi: 10.1128/IAI.72.6.3391-3397.2004 15155645 10.1128/IAI.72.6.3391-3397.2004 PMC415716

[55] Gandhi NA, Bennett BL, Graham NM, Pirozzi G, Stahl N, Yancopoulos GD (2016) Targeting key proximal drivers of type 2 inflammation in disease. Nat Rev Drug Discov 15: 35–50. doi: 10.1038/nrd4624 26471366 10.1038/nrd4624

[56] Jakubzick C, Choi ES, Joshi BH, Keane MP, Kunkel SL, Puri RK, Hogaboam CM (2003) Therapeutic attenuation of pulmonary fibrosis via targeting of IL-4- and IL-13-responsive cells. J Immunol 171: 2684–2693. 12928422 10.4049/jimmunol.171.5.2684

[57] McGaha TL, Le M, Kodera T, Stoica C, Zhu J, Paul WE, Bona CA (2003) Molecular mechanisms of interleukin-4-induced up-regulation of type I collagen gene expression in murine fibroblasts. Arthritis Rheum 48: 2275–2284. doi: 10.1002/art.11089 12905482 10.1002/art.11089

[58] Peng H, Sarwar Z, Yang XP, Peterson EL, Xu J, Janic B, Rhaleb N, Carretero OA, Rhaleb NE (2015) Profibrotic Role for Interleukin-4 in Cardiac Remodeling and Dysfunction. Hypertension 66: 582–589. doi: 10.1161/HYPERTENSIONAHA.115.05627 26195478 10.1161/HYPERTENSIONAHA.115.05627 PMC4685692

[59] Bergman I, Loxley R (1970) New spectrophotometric method for the determination of proline in tissue hydrolyzates. Anal Chem 42: 702–706. 5431521 10.1021/ac60289a036

