# Supplementary figures and images for: Host regulation of liver fibroproliferative pathology during experimental schistosomiasis via interleukin-4 receptor alpha
Source: PLoS Negl Trop Dis. 2017 Aug 21;11(8):e0005861. doi: 10.1371/journal.pntd.0005861 (PMC5578697; doi:10.1371/journal.pntd.0005861)

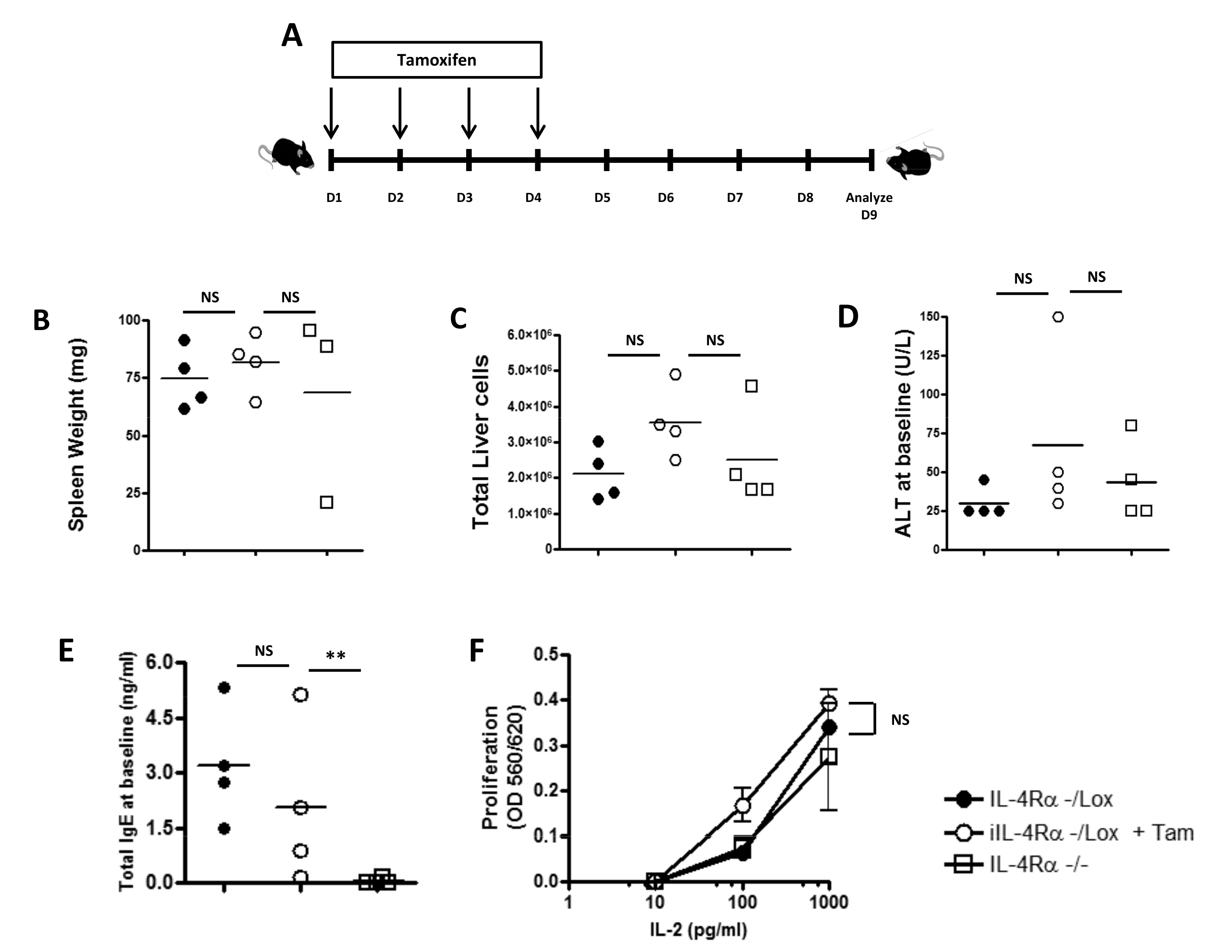

Supplement: S1 Fig — A. Experimental design. B. Spleen weights. C. Total liver cell numbers. D. Hepatocellular damage at baseline. Alanine transaminase (ALT) sera concentration. E. Total seric IgE at baseline. F. Unimpaired IL-2-mediated proliferation of splenocytes in the absence of IL-4Rα. Splenocytes from control (IL-4Rα-/lox), Tamoxifen-treated iCre-/+ IL-4Rα-/lox mice and IL-4Rα-/- mice were stimulated with IL-2. Metabolic activity was measured by colorimetric detection of resazurin reduction into resofurin. Mean ± SD of triplicate cultures; NS = p > 0.05; * = p < 0.05; ** = p < 0.01; *** =, p < 0.001; **** = p < 0.0001. (TIF) [file pntd.0005861.s001.tif]

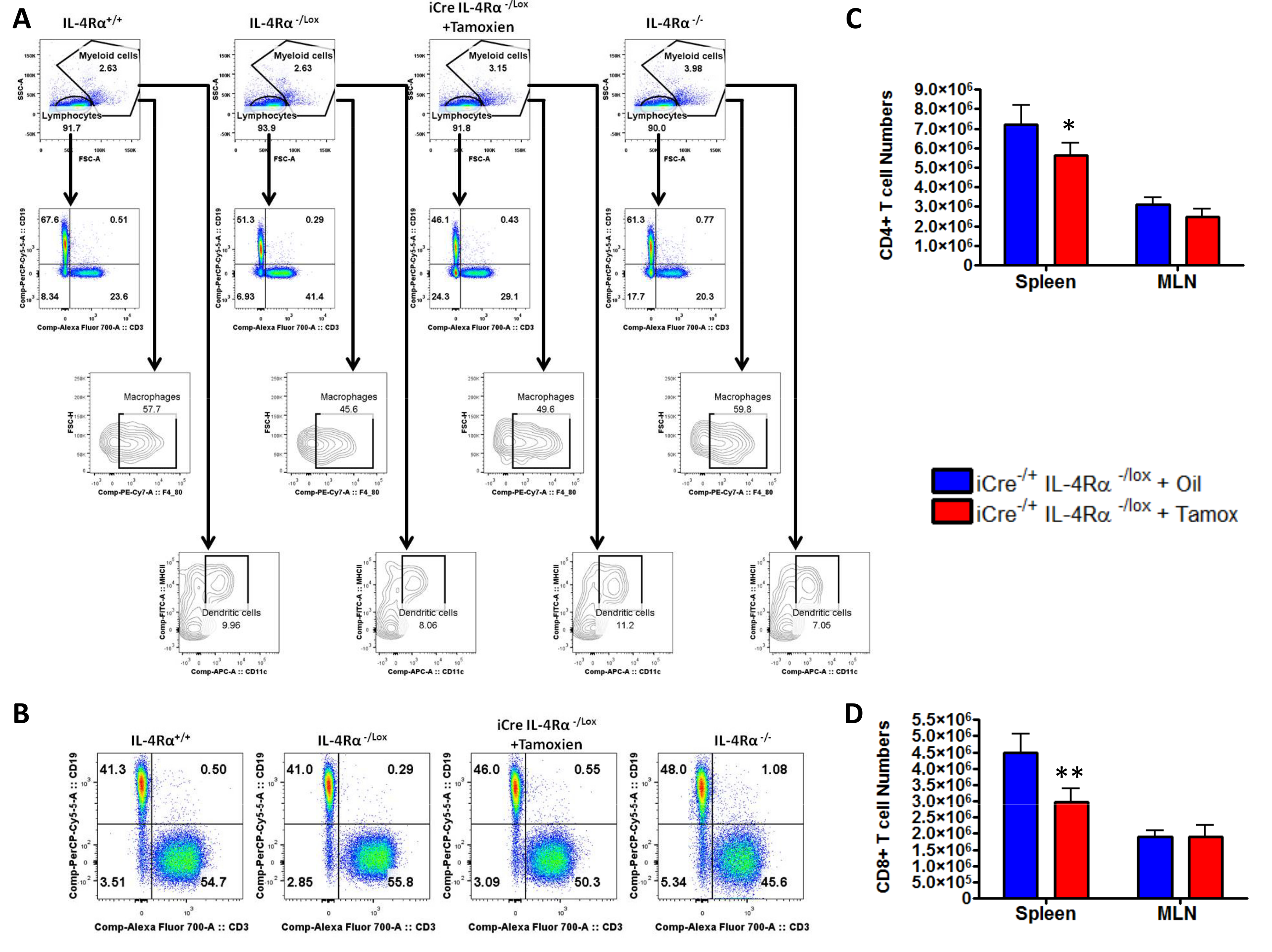

Supplement: S2 Fig — A. Gating strategy and average frequencies of CD3+ T cells, CD3-CD19+ B cells, F4/80+ macrophages and CD11c+MHCII+ dendritic cells in the spleen of wt (IL-4Rα+/+), littermate controls (IL-4Rα-/lox), Tamoxifen-fed iCre-/+IL-4Rα-/lox and IL-4Rα-deficient (IL-4Rα-/-) mice. B. Frequencies of CD3+ T cells, CD3-CD19+ B cells, in the MLN of wt (IL-4Rα+/+), littermate controls (IL-4Rα-/lox), Tamoxifen-fed iCre-/+IL-4Rα-/lox and IL-4Rα-deficient (IL-4Rα-/-) mice. Scatter plots are representative of analyses performed at least twice with 3–4 mice per group. Total CD4+ (C) and CD8+ (D) T cell numbers in the MLN of naive iCre-/+ IL-4Rα-/lox mice 5 days following treatment. (TIF) [file pntd.0005861.s002.tif]

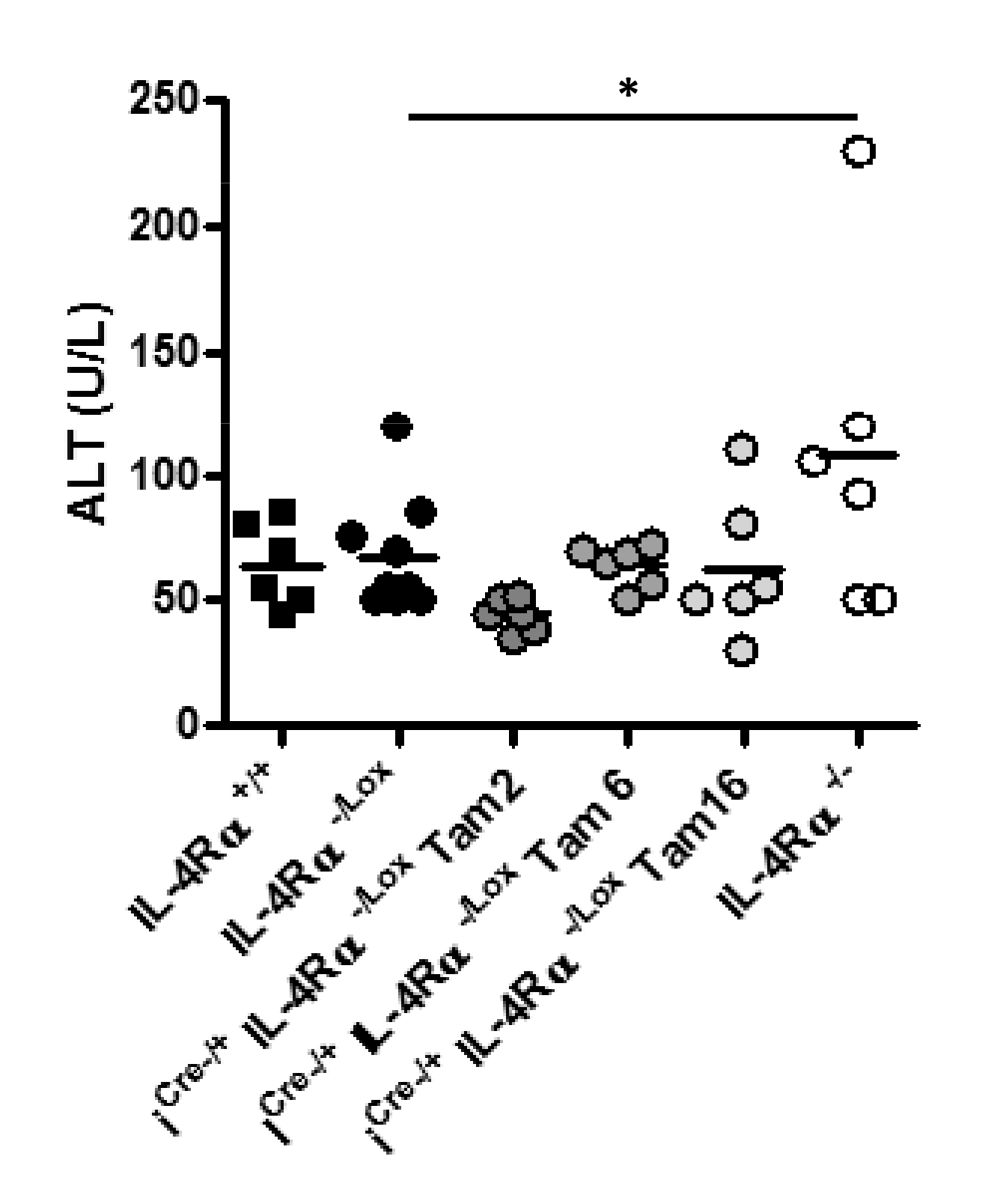

Supplement: S3 Fig — Alanine transaminase (ALT) sera concentration 18 weeks post-infection with S. mansoni. Experiment was conducted twice with 5–10 mice per group. Data are expressed as mean ± SD; NS = p > 0.05; * = p < 0.05. (TIF) [file pntd.0005861.s003.tif]

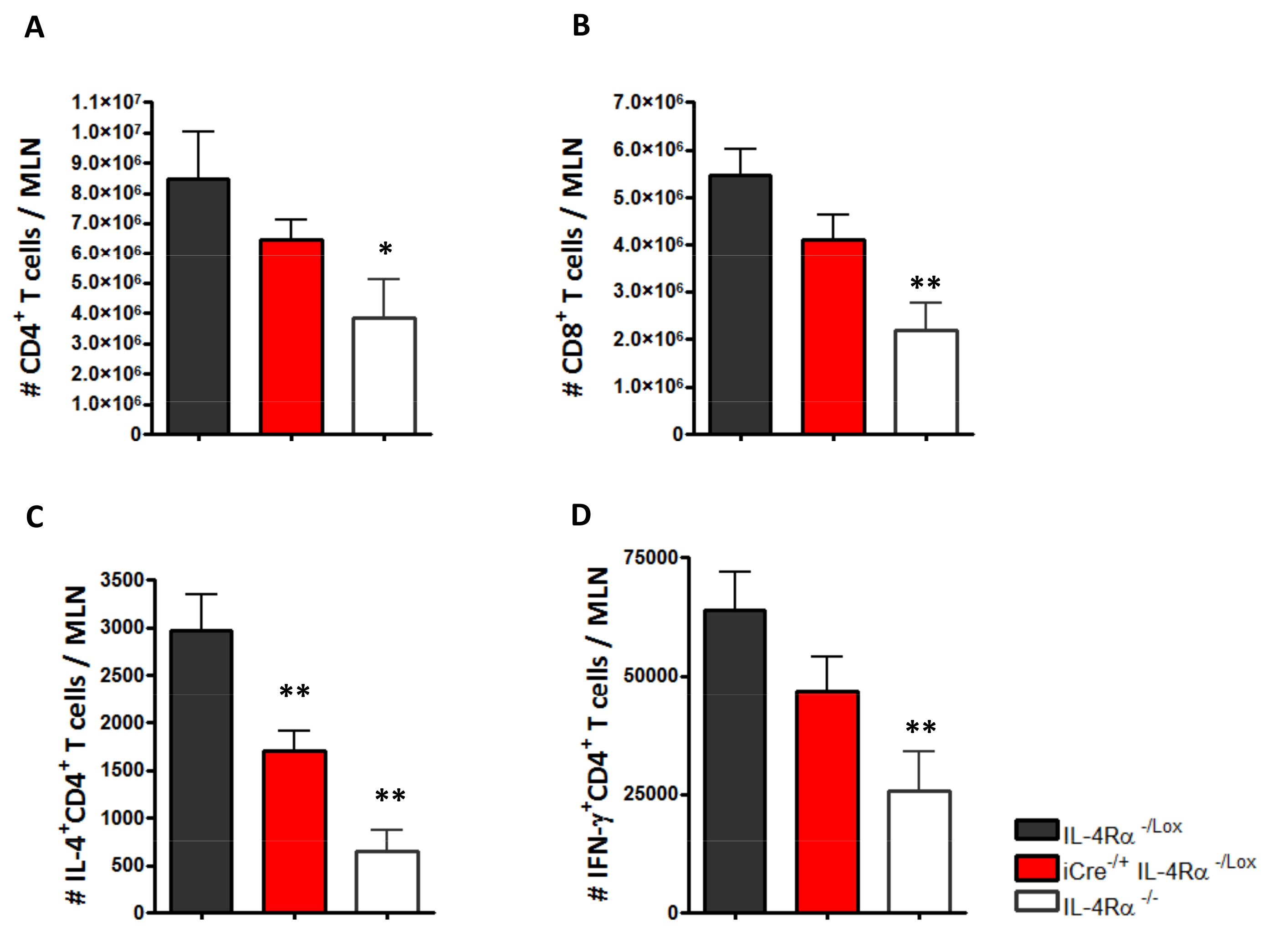

Supplement: S4 Fig — A. Total MLN CD4+ T cell numbers. B. Total MLN CD8+ T cell numbers. Total IL-4-producing (C) and IFNγ-producing (D) MLN CD4+ T cell numbers. Each experiment was conducted at least twice with 5–10 mice per group. Data are expressed as mean ± SD; NS = p > 0.05; * = p < 0.05; ** = p < 0.01; *** =, p < 0.001; **** = p < 0.0001. (TIF) [file pntd.0005861.s004.tif]

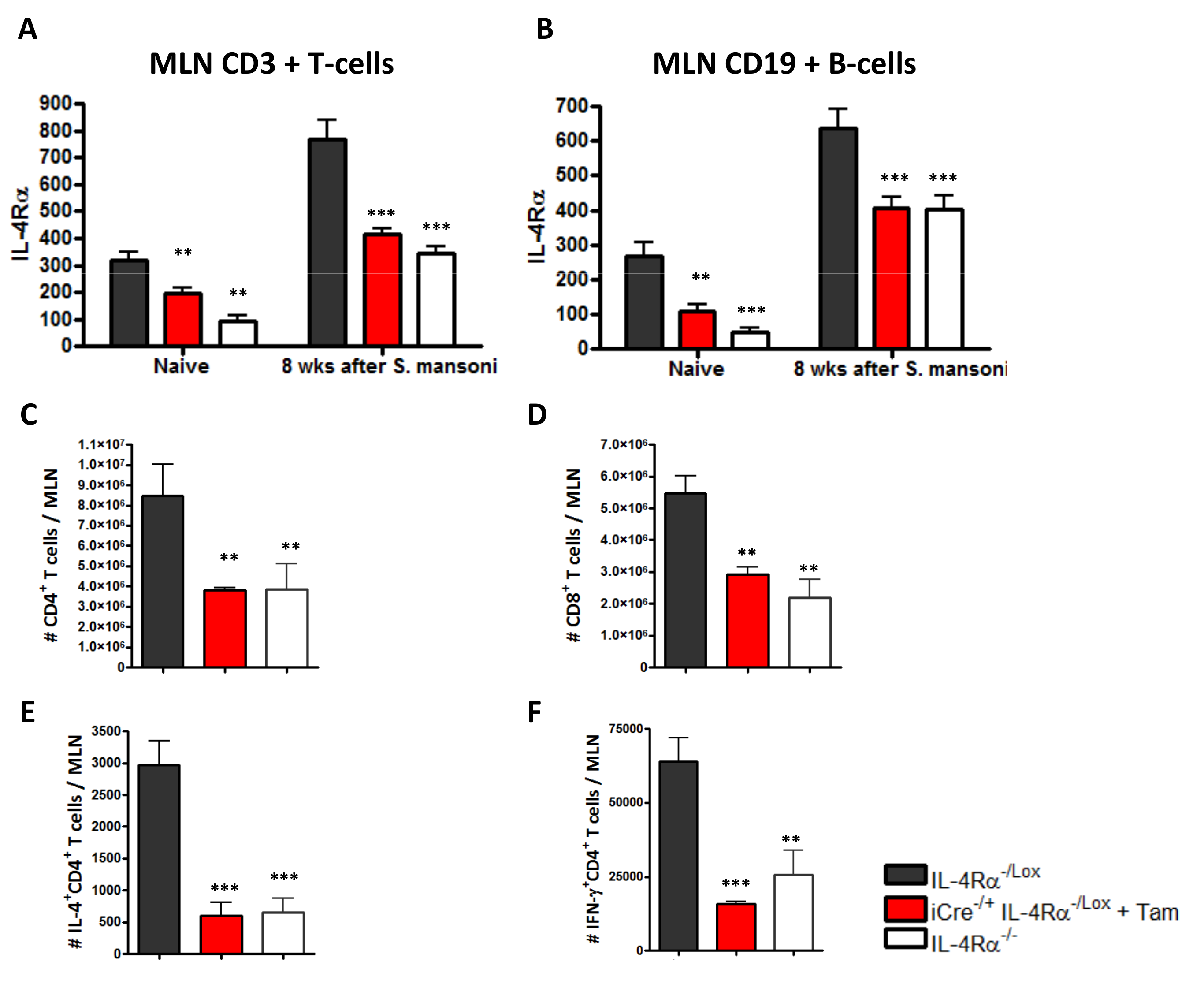

Supplement: S5 Fig — IL-4Rα GMFI on CD3+CD4+ (A) T cells and CD3-CD19+ B cells (B) from MLN of naïve (collected 5 days following Tamoxifen treatment) vs. MLN of Tam6 S. mansoni-infected mice. C. Total Tam6 MLN CD4+ T cell numbers. D. Total Tam6 MLN CD8+ T cell numbers. Total IL-4-producing (E) and IFNγ-producing (F) MLN CD4+ T cell numbers under the Tam6 scheme. Each experiment was conducted at least twice with 5–10 mice per group. Data are expressed as mean ± SD; NS = p > 0.05; * = p < 0.05; ** = p < 0.01; *** =, p < 0.001. (TIF) [file pntd.0005861.s005.tif]

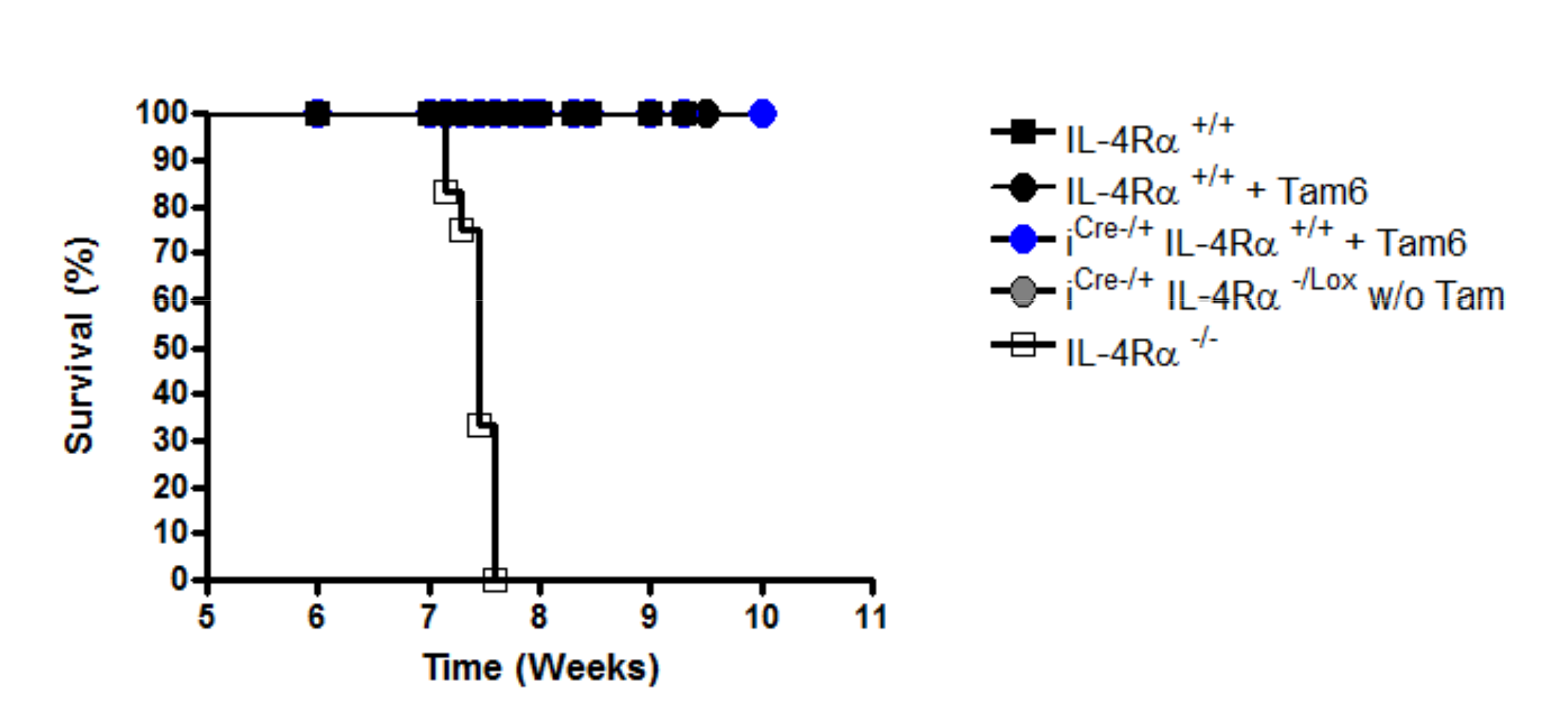

Supplement: S6 Fig — Mice (10 per group) were infected percutaneously with 80 S. mansoni cercariae and monitored over time. Presented are the weekly percentages of survivors. (TIF) [file pntd.0005861.s006.tif]

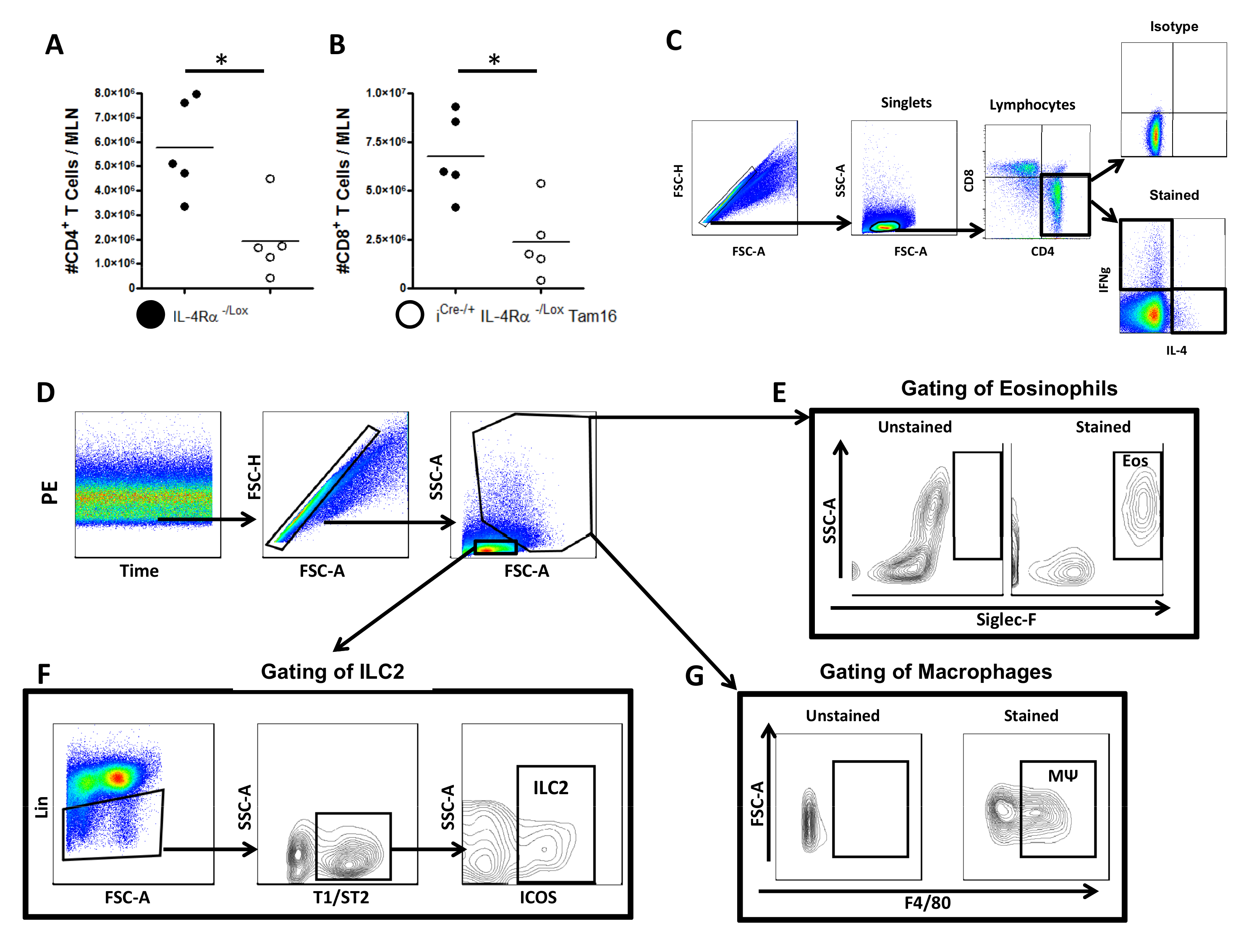

Supplement: S7 Fig — A. Total Tam16 MLN CD4+ T cell numbers. B. Total Tam16 MLN CD8+ T cell numbers. C. Gating of cytokine-producing CD4+ T cells. D. Gating within MLN lymphoid and myeloid cells to define Sschi SiglecF+ eosinophils (E), Lin-T1/ST2+ICOS+ ILC2 (F) and Fschi F4/80+ macrophages,(G). (TIF) [file pntd.0005861.s007.tif]

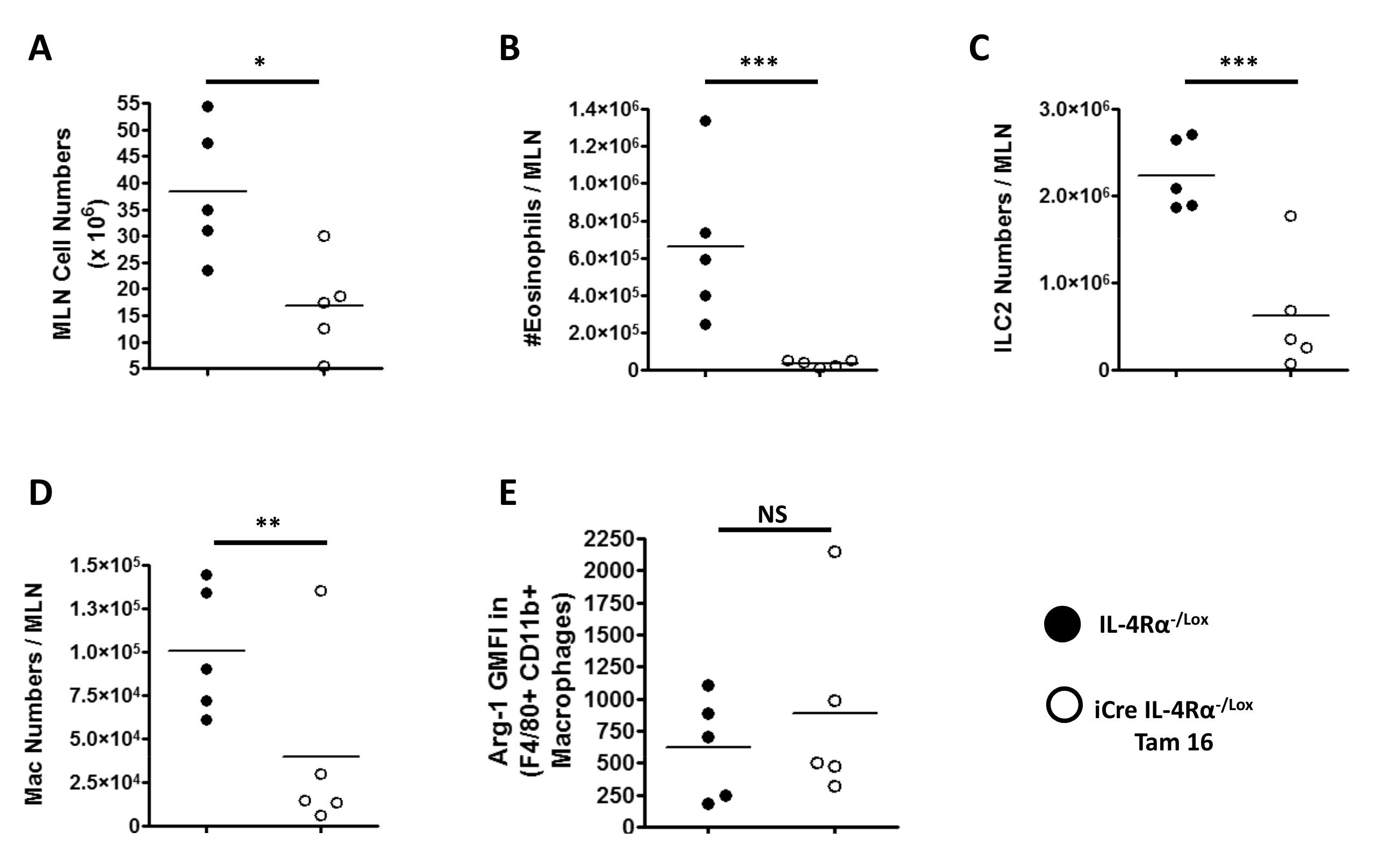

Supplement: S8 Fig — Changes in total numbers of MLN cells (A), Eosinophils (B), ILC2 (C), macrophages (D) and Arginase expression by macrophages (E) in S. mansoni-infected mice following IL-4Rα knockdown at 16 weeks post-infection. Each experiment was conducted at least twice with 3–6 mice per group. Data are expressed as mean ± SD; NS = p > 0.05; * = p < 0.05; ** = p < 0.01; *** =, p < 0.001; **** = p < 0.0001. (TIF) [file pntd.0005861.s008.tif]
